# Induction of expression of aryl hydrocarbon receptor-dependent genes in human HepaRG cell line modified by shRNA and treated with β-naphthoflavone

**DOI:** 10.1007/s11010-016-2862-3

**Published:** 2016-10-28

**Authors:** Damian Brauze, Piotr Zawierucha, Katarzyna Kiwerska, Kinga Bednarek, Martyna Oleszak, Malgorzata Rydzanicz, Malgorzata Jarmuz-Szymczak

**Affiliations:** 1 Institute of Human Genetics, Polish Academy of Sciences, Strzeszynska 32, 60-479 Poznan, Poland; 2 Department of Histology and Embryology, Poznan University of Medical Sciences, 60-781 Poznan, Poland; 3 Department of Anatomy, Poznań University of Medical Sciences, 60-781 Poznan, Poland; 4 Department of Medical Genetics, Medical University of Warsaw, Pawinskiego 3c, 02-106 Warsaw, Poland

**Keywords:** *AhR*, *CYP1A1*, *CYP1A2*, *CYP1B1*, *CYP19A1 SERPINB2*, *STC2*, *SLC7A5*, *CCNE2*, *NQO1*, *GSTA2*, TIPARP, ARL4C, β-Naphthoflavone, HepaRG cell line

## Abstract

The aryl hydrocarbon receptor (AhR) mediates a variety of biological responses to ubiquitous environmental pollutants. In this study, the effects of administration of β-naphthoflavone (BNF), a potent AhR ligand, on the expression of AhR-dependent genes were examined by microarray and qPCR analysis in both, differentiated and undifferentiated HepaRG cell lines. To prove that BNF-induced changes of investigated genes were indeed AhR-dependent, we knock down the expression of *AhR* by stable transfection of HepaRG cells with shRNA. Regardless of genetical identity, our results clearly demonstrate different expression profiles of AhR-dependent genes between differentiated and undifferentiated HepaRG cells. Genes involved in metabolism of xenobiotics constitute only minute fraction of all genes regulated by AhR in HepaRG cells. Participation of AhR in induction of expression of genes associated with regulation of apoptosis or involved in cell proliferation as well as AhR-dependent inhibition of genes connected to cell adhesion could support suggestion of involvement of AhR not only in initiation but also in progression of carcinogenesis. Among the AhR-dependent genes known to be involved in metabolism of xenobiotics, cytochromes *P4501A1* and *1B1* belong to the most inducible by BNF. On the contrary, expression of *GSTA1* and *GSTA2* was significantly inhibited after BNF treatment of HepaRG cells. Among the AhR-dependent genes that are not involved in metabolism of xenobiotics *SERPINB2*, *STC2*, *ARL4C*, and *TIPARP* belong to the most inducible by BNF. Our results imply involvement of Ah receptor in regulation of *CYP19A1*, the gene-encoding aromatase, and an enzyme responsible for a key step in the biosynthesis of estrogens.

**Electronic supplementary material:**

The online version of this article (doi:10.1007/s11010-016-2862-3) contains supplementary material, which is available to authorized users.

## Introduction

The aryl hydrocarbon receptor is a ligand-dependent transcription factor that mediates a variety of biological responses to ubiquitous environmental pollutants such as polycyclic aromatic hydrocarbons (PAH) and chlorinated dibenzo-*p*-dioxins [1]. Despite the variability observed between experiments aiming to discover AhR-dependent genes, a small subset of AhR target genes, including *CYP1A1*, *CYP1A2*, *CYP1B1*, *NQO1*, *ALAH3A1*, *UGT1A*, and *GSTA1*, are commonly upregulated following AhR activation. These genes encode phase I and phase II xenobiotic-metabolizing enzymes, which function to metabolize activating compounds and thus provide a vital role in the detoxification of xenobiotics [2–5]. These enzymes metabolize many of their substrates to more soluble and excretable products, but as the classic example of benzo[a]pyrene shows, the same enzymes are also responsible for activation of substrates to ultimate carcinogenic metabolites. This leads to DNA adducts formation, induction of sister chromatid exchanges and carcinogenesis [6–9]. Experiments with knockout animals revealed that PAH-induced carcinogenicity is lost in *AhR*-deficient mice [10]. Moreover, functional analysis of *AhR* knockout mice revealed that AhR is involved in lethality, teratogenesis, immunotoxicity, hepatotoxicity, and tumor promotion caused by 2,3,7,8-tetrachlorodibenzo-*p*-dioxin (TCDD) [11–15].

AhR resides in the cytoplasm as a complex with chaperone proteins: HSP90, XAP2, and p23 [16]. The receptor binds to xenobiotics such as β-naphthoflavone (BNF), benzo[a]pyrene, 3-methylcholanthrene, or TCDD with high affinity. Subsequently, the AhR ligand complex translocates to the nucleus, where after dissociation of chaperone proteins, it binds to AhR nuclear translocator (ARNT) protein [17, 18]. Then, the liganded AhR/ARNT heterodimer binds to xenobiotics responsive element sequences (XRE), which constitute enhancer DNA elements present in the 5′-flanking region of target genes. Elevated expression of target genes leads to altered metabolism, which often results in enhanced carcinogenesis and toxicity [19]. Activation of procarcinogenic PAHs to ultimate carcinogens by AhR regulating enzymes is traditionally considered as the first step in tumor initiation. On the other hand, numerous studies have shown that the AhR plays a role not only in tumor initiation but also in promotion and progression [20, 21]; however molecular mechanisms involved in these processes are not fully understood. Some pleiotropic effects of AhR activation could be partially explained by cross-talk with other signal transduction pathways. The ability of AhR agonists to interfere with multiple signal transduction pathways, including those regulated by nuclear receptors, has been reported by many laboratories and involves multiple mechanisms [22–26]. Although the best studied AhR-responsive genes produce enzymes involved in xenobiotics metabolism, gene expression profiling studies have identified a large number of other genes that are induced or repressed in an AhR- and ligand-dependent manner [27–33]. But a majority of discussed studies were accomplished with application of laboratory animals. However, substantial differences in regulation of AhR-dependent genes between human and mouse were reported [34].

The present study was therefore designed to investigate the role of AhR in BNF-regulated gene expression in HepaRG cells. BNF, a well-known AhR agonist [35], is a widely used inducer of phase I and phase II enzymes in xenobiotic metabolism which is considered as not being cancerogenic unlike majority of other PAHs [36, 37]. BNF has been also shown to suppress chemical carcinogenesis at numerous sites in mice [38]. In this report, we examined the microarray-based expression profiles of AhR-dependent genes. HepaRG cells, derived from a human hepatocellular carcinoma, exhibit unique features: when seeded at low density, they acquire an elongated undifferentiated morphology and actively divide, but after having reached confluency in the presence of DMSO, they form typical hepatocyte-like colonies surrounded by biliary epithelial-like cells. Moreover, contrary to other human hepatoma cell lines, HepaRG cells express various CYPs and the nuclear receptors constitutive androstane receptor (CAR) and pregnane X receptor (PXR) at levels comparable to those found in cultured primary human hepatocytes. They also express other genes with various functions, such as phase 2 enzymes, solute carrier transporters, albumin, haptoglobin as well as aldolase B that is a specific marker of adult hepatocytes [39]. The expression of AhR-dependent genes was found to be similar in highly differentiated HepaRG cells and in primary human hepatocytes [40]. Likewise, our earlier experiments revealed a diverse expression of some AhR-dependent genes in undifferentiated and differentiated HepaRG cells [41]. It was demonstrated that AhR was highly expressed in developing fetal liver of mouse embryo and presumably involved in liver development [42]. However, different cell types were involved in AhR-dependent development of liver and in AhR-dependent hepatotoxicity [43]. It seems to be plausible that undifferentiated HepaRG cells are equivalent to cells from fetal liver of mouse embryo, whereas differentiated ones resemble maturated hepatocytes. Therefore, HepaRG cell line gives us opportunity to investigate AhR-dependent regulation of genes in the cells with identical DNA but committed to diverse development programs. Consequently, we decided to compare the expression profiles of AhR-dependent genes in both stages of HepaRG cell differentiation. To prove that BNF-induced changes of investigated genes were indeed AhR-dependent, we knocked down the expression of *AhR* by stable transfection of HepaRG cells with shRNA. Quantitative PCR of the most interesting candidate genes was performed to validate the microarray results.

## Materials and methods

### Chemicals

BNF, SYBR^®^ Green I (10,000× concentration), agarose, JumpStart Taq DNA polymerase, Enhanced Avian RT first-strand synthesis kit (STR-1), GenElute™ PCR Clean-Up Kit, GenElute™ HP Endotoxin-Free Plasmid Maxiprep Kit, PCR Low Ladder Marker Set, guanidine thiocyanate, ammonium thiocyanate, Williams’ E medium, LB broth, and LB agar were supplied by Sigma-Aldrich Co (St. Louis, MO, USA). Fluorescein was obtained from Bio-Rad Laboratories (Hercules, CA, USA). Restriction endonucleases were purchased from Fermentas International Inc. (Burlington, Canada). Deoxyribonucleotide triphosphates such as dATP, dGTP, aCTP, and dTTP were provided by Roche Diagnostics (Mannheim, Germany). PCR primers were provided by Institute of Biochemistry and Biophysics, Polish Academy of Sciences, Poland (oligo.pl), and Genomed, Poland. Agilent RNA 6000 Reagents were provided by Agilent Technologies (Santa Clara, CA, USA). Affymetrix Human Genome U219 Array Strip, GeneChip 3′IVT Express Kit and GeneAtlas Hybridization, and Wash and Stain Kit for 3′IVT Arrays were provided by Affymetrix (Santa Clara, CA, USA). GeneClip™ U1 Hairpin Cloning System—Neomycin Vector and antibiotic G418 (Geneticin)—was provided by Promega (Madison, WI, USA). Lipofectamine 2000 and Opti-MEM^®^ I Reduced Serum Medium was provided by Invitrogen (Carlsbad, CA, USA). All the other compounds were readily available as commercial products.

### HepaRG cell line and BNF treatment

HepaRG cells were obtained from Biopredic Ltd. (Rennes, France). The procedures of plating and maintaining HepaRG cells were described previously [44]. In brief, HepaRG cells were cultured in 25-cm^2^ flasks (37 °C, 5% CO_2_) either in Williams’ E medium supplemented with 10% FBS, 100 units/ml penicillin, 100 µg/ml streptomycin, 5 µg/ml insulin, 2 mM glutamine, and 5 × 10^−5^ M hydrocortisone hemisuccinate (undifferentiated cells) or, after reaching full confluence in differentiation medium corresponding to the above one, but supplemented with 2% of DMSO (differentiated cells).

HepaRG cell line was treated with BNF dissolved in DMSO to a final concentration of 100 µM in medium (8 µl 50 mM BNF/4 ml medium in 25-cm^2^ flask; 0.2% of DMSO) for 24 h. Appropriate amount of solvent (DMSO) were added to control, untreated cells.

### GeneClip hairpin oligonucleotide design and transformation of *Escherichia coli*

Selection of siRNA hairpin target sequence to AhR mRNA (NM_001621) was achieved by using siDESIGN Center (http://dharmacon.gelifesciences.com/design-center/?redirect=true) and the rules from technical manual from GeneClip™ U1 Hairpin Cloning System (Promega). We have designed four different siRNA targets, from which the best one was selected for the subsequent analysis. Selected oligonucleotides (siRNA target underlined) purchased from “oligo.pl” were as follows: forward-5′-TCTCGAACAGAGCATTTACGAAATTCAAGAGATTTCGTAAATGCTCTGTTCCT-3′ and reverse-5′-CTGCAGGAACAGAGCATTTACGAAATCTCTTGAATTTCGTAAATGCTCTGTTC-3′.

Sequences were hybridized and ligated to pGeneClip neomycin vector construct according to the manufacturer's instruction. Further, One Shot^®^ TOP10 competent *E. coli* cells (Invitrogen) were transformed with the vector and cloned. The pGeneClip vector was isolated back from bacteria by GenElute™ HP endotoxin-free plasmid maxiprep kit and digested with *Pst*I to confirm the presence of an appropriate insert.

As a negative control for RNA interference, a nonspecific target sequence (scrambled) was used. Nonspecific sequence was designed, ligated to plasmid, and multiplicated into bacteria in the same way as presented above. Sequences of nonspecific oligonucleotides were as follows: forward-5′-TCTCGTAGTAGGCATCGACATATTTCAAGAGAATATGTCGATGCCTACTACCT-3′ and reverse-5′-CTGCAGGTAGTAGGCATCGACATATTCTCTTGAAATATGTCGATGCCTACTAC-3′. This nonspecific sequence was not complementary to any known human, rat, and mouse sequence.

### Transfection of pGeneClip vector to HepaRG cells

Stable transfection of pGeneClip neomycin vector construct to HepaRG cells was carried out according to the suggestions from GeneClip™ U1 Hairpin Cloning System's technical manual (Promega). We have applied lipofection with Lipofectamine 2000 (Invitrogen) as transfection method. Cell line cultures were grown as monolayer in 500 µl of Williams’ E medium supplemented with 10% FCS (without standard antibiotics) to reach about 90% confluence of flask bottom (2 cm^2^). Liposomes were prepared by mixing 0.8 µg plasmid DNA diluted in 50 µl Opti-MEM medium with 2 µl of Lipofectamine 2000 in 50 µl of Opti-MEM. Following incubation for 20 min, DNA-lipid complexes (100 µl) were added to HepaRG cells. Cells were passaged at 1:10 dilution into fresh growth medium with standard antibiotics 24 h after transfection. Selective antibiotic G418 was added to the medium 3–4 days later, when cells reached about 50% confluence.

### RNA isolation

Total RNA was isolated directly from monolayer cells in culture dish as described before [45]. The extracted total RNA dissolved in water was quantified spectrophotometrically at 260 nm (*A*_260_; NanoDrop). The *A*_260/280_ ratio > 1.9 was considered as an acceptable measurement of RNA purity. RNA integrity was estimated by BioAnalyzer 2100 analysis (Agilent, RIN: 9.40–9.80). The amount of cDNA synthesized in a single reaction was sufficient to PCR-amplify all genes.

### Microarray-based gene expression analysis and statistics

Expression analysis was performed using Human Genome U219 array (Affymetrix) in duplicate biological replicates of each sample type.

#### RNA preprocessing

cDNA was synthetized in two steps, namely first-strand synthesis and second-strand synthesis, respectively, using Affymetrix GeneChip^®^ 3′IVT Express Kit (Affymetrix, Santa Clara, CA, USA) according to the manufacturer’s instructions. Biotin-labeled cRNA synthesis (IVT Labeling) and cRNA fragmentation were performed by Affymetrix GeneChip^®^ kit reagents according to the procedure described in the Affymetrix GeneAtlas™ 3′IVT Express Kit's technical manual.

#### Target hybridization and scanning

Biotin-labeled and fragmented target cRNA samples were loaded into Affymetrix GeneChip^®^ (Human Genome U219) Array Strip together with control cRNAs and oligo B2. Hybridization procedure was conducted at 45 °C, for 16 h in AccuBlock™ Digital Dry Bath (Labnet international, Inc.) hybridization oven. Washing and staining procedure was performed in Affymetrix GeneAtlas™ Fuidics Station according to the instructions in the technical manual. Affymetrix GeneAtlas™ Imaging Station was used for scanning the arrays.

#### Data analysis and preparation of gene lists

Preliminary analysis of the scanned chips was performed using Affymetrix GeneAtlas™ Operating Software. The quality of gene expression data was checked according to quality control criteria provided by the software. Then, Partek^®^ Express™ Software (Partek, Inc., Chesterfield, MO, USA) was used for further data analysis and evaluation. Using quality control checkpoints and statistical analysis of gene fold-change significances, table of the most important changes in gene expression was created. Next, generated table was imported to Pathway Studio^®^ Explore (Ariadne Genomics, Rockville, MD, USA) where right statistical analyses were made. To evaluate the *P* value indicating the significance of the enrichment score, nonparametric Mann–Whitney statistical test was used (*α* = 0.05). Venn diagrams were calculated and drawn using online software tool—(http://bioinformatics.psb.ugent.be/webtools/Venn/). Selected list of genes was further analyzed by online DAVID functional annotation tools (https://david.ncifcrf.gov/home.jsp) [46].

### Real-time PCR-based gene expression analysis and statistics

Expression analysis was performed using two-step quantitative real-time PCR with SYBR^®^ Green I chemistry in triplicate biological replicates of each sample type.

#### cDNA synthesis for real-time PCR

Eight micrograms of total RNA was reverse-transcribed to cDNA in a total volume of 40 µl, using Enhanced Avian RT first-strand synthesis kit (Sigma-Aldrich Co., St. Louis, MO, USA) according to the manufacturer’s instruction. Random nonamers were used as primers of the reaction. The amount of cDNA synthesized in a single reaction was sufficient to PCR-amplify all genes (targets and standards).

#### Primer design for real-time PCR

PCR primers were designed according to published genes sequences (GenBank, accession numbers in Table 1) with the Beacon Designer™ software (PRIMER Biosoft International) and their specificity was verified with BLAST alignment search (http://blast.ncbi.nlm.nih.gov/Blast.cgi). Primers and/or amplicons were designed to cross the exon/exon boundaries (Table 1). To confirm amplification of the expected size fragment, amplification products were characterized by agarose gel electrophoresis. Identity of amplicons was further verified by the analysis of digestion products generated by restriction endonucleases (not shown). Primers for reference genes and other genes used herein were published earlier [41].

**Table 1:** Sequence of primers used in real-time PCR, amplicon sizes, annealing temperatures, and the amplification efficiencies

Target's Accession No.	Sequences	Amplicon length (bp)	Annealing Tm(^o^C)/t(s)	PCR efficiency
SERPINB2 NM_002575	F: 5′ GAATGCTGTCTACTTCAA 3′	147-i	55/15	0.99
	R: 5′ TCTTCTATGTATCCAATGTT 3′			
SLC7A5 NM_003486	F: 5′ G-GTGATGTGTCCAATCTA 3′	116	56/15	1.00
	R: 5′ AAGTAATTC-CATCCTCCATA 3′			
SLC14A1 NM_015865	F: 5′ GACATTACAATCCATTCT 3′	140-i	52/15	0.96
	R: 5′ ATTATCACAGCCATAGAT 3′			
CCNE2 NM_057749	F: 5′ GTTCTTCTACCTCAGTATTCTC 3′	114-i	55/10	0.98
	R: 5′ AGCAGCAGTCAGTATTCT 3′			
TIPARP NM_015508	F: 5′ CTGTCTTGCCATATCATT 3′	144	55/10	0.96
	R: 5′ ATTCTTGTCC-TCCATACT 3′			
STC2 NM_003714	F: 5′ CAACTCTTGTGAGATTCG 3′	110-i	58/15	0.91
	R: 5′ TACATTTCAAGGCGTCTT 3′			
SCG5 NM_003020	F: 5′ CAAGAAACTCCTTTACGA 3′	138-i	56/15	0.93
	R: 5′ TCCTTATCCTCATCTGAA 3′			
TMEM156 NM_024943	F: 5′ G-TTCTTATCAGGAGAGGAT 3′	123	56/15	0.94
	R: 5′ ATGACAGGTAGTGTTATATTC 3′			
GSTA2 NM_000846	F: 5′ CCACTACTCCAATATACG 3′	165-i	58/15	0.94
	R: 5′ CCATCAATCTCAACCATT 3′			

The hyphen in the primer sequence denotes the exon/exon boundary. Letter “i” after the amplicon length indicates that exon/exon boundary was inside the amplified sequence

#### Real-time PCR

cDNA of investigated genes was amplified by real-time PCR in the iCycler iQ5 real-time PCR detection system with iQ5 optical system software 2.0 (Bio-Rad Laboratories; Hercules, CA, USA) using SYBR^®^ Green I as the detection dye. Amplification was carried out in a total volume of 20 µl containing 0.2x SYBR^®^ Green I, PCR buffer (50 mM KCl, 10 mM Tris–HCl, pH 8.3), 3.5 mM MgCl_2_, 10 nM fluorescein, 0.2 µM each primer, 0.2 mM each dNTPs, 0.5 U JumpStart Taq DNA polymerase, and 0.4 µl cDNA (undiluted reverse-transcription product derived from 8 µg RNA in 40 µl reaction). The reactions were cycled 40 times using the following parameters: 95 °C for 10 s, 52–60 °C for 5–15 s (Table 1 plus [41]), and 72 °C for 15–20 s during which the fluorescence data were collected. At the end of the PCR, a melting curve was generated by heating the samples from 50 to 95 °C in 0.5 °C increments with a dwell time at each temperature of 10 s to verify the specificity of the product. Nontemplate controls were run with every assay and no indication of PCR contamination was observed. Lack of PCR products from the nonreverse transcribed RNA control indicated that possible contamination of the genomic DNA has not served as an amplification template.

#### Quantitative PCR data analysis and statistics

Expression levels of the target genes were normalized with respect to two reference genes, *ß*-*actin* and *ARNT*, using relative quantification method. The *ß*-*actin* expression was reported as not affected by treatment of rats with TCDD [47]. The *ARNT* mRNA levels were not altered as the result of in vivo treatment of rats with TCDD, 3-MC, and BNF [48]. Similar observation was reported by Franc et al. [49] with rats exposed to TCDD. All calculations were performed using Gene Expression Macro™ 1.10 software (Bio-Rad Laboratories, CA, USA).

To determine the limit of detection and the efficiency of PCR amplification of reference and target genes, dilution series (1:5 dilution) of PCR products were prepared. PCR products (about 200 µl) were purified using EZ-10 Spin Column PCR purification Kit (Bio Basic Inc., Canada). Concentration of DNA was determined spectrophotometrically (NanoDrop) and the number of copies of a template was calculated using online software (http://www.uri.edu/research/gsc/resources/cndna.html). Molecular biology-grade tRNA from *E. coli* (100 ng/µl) was used as a carrier during dilutions. Each dilution was amplified in triplicate by real-time PCR and the obtained quantification cycle (*C*_q_) values were used to construct a graph *C*_q_ vs. log10 of the number of template copies. The slope of the graph was used to determine the reaction efficiency according to the formula: Efficiency = [10^(−1/slope)^] − 1. The efficiencies of target and reference genes amplification are shown in Table 1. Limit of detection defined as minimal number of DNA copies that can be detected with reasonable certainty was estimated as 10 copies per single PCR reaction (*C*_q_ about 33–36) for reference and target genes. We were capable to observe amplification even in a single template copy PCR reaction, but due to stochastic processes such detection was rather qualitative than quantitative.

The relative gene expression was calculated for the triplicate samples derived from each RT reaction by Gene Expression MacroTM 1.10 (Bio-Rad) software. The average of the three values was carried forward as the value to be entered into calculation of the mean ± SD for each treatment group.

Statistical significance of differences was assessed by one-way ANOVA followed by Tukey–Kramer post-test (Table 8). All calculations were done using GraphPad Prism for Windows version 6.01 (GraphPad Software, San Diego CA); *P* ≤ 0.05 was considered statistically significant.

### Experimental design

We have used the expression microarrays and real-time PCR to study the transcriptional response to 100 µM BNF in transfected control and AhR(−) HepaRG cells. Control HepaRG cell line was stably transfected with unspecific construct, whereas AhR(−) HepaRG line was transfected with construct silencing *AhR* expression by RNA interference. Experiments were performed on both, undifferentiated and differentiated HepaRG cell lines (Table 2). The effects of BNF on the expression of AhR-dependent genes were evaluated 24 h after administration of BNF. Solvent-treated, AhR(−)-transfected HepaRG cells, both undifferentiated and differentiated, were analyzed only by real-time PCR.

**Table 2:** Summary of the experimental design applied to analyze gene expression by microarray and qPCR

Type of cells	Transfected vector	Treatment
HepaRG undifferentiated	Negative control	DMSO
HepaRG undifferentiated	Negative control	BNF
*HepaRG undifferentiated*	*AhR(*−*)*	*DMSO*
HepaRG undifferentiated	AhR(−)	BNF
HepaRG differentiated	Negative control	DMSO
HepaRG differentiated	Negative control	BNF
*HepaRG differentiated*	*AhR(*−*)*	*DMSO*
HepaRG differentiated	AhR(−)	BNF

The negative control shRNA is a scrambled artificial sequence which does not match any human gene. The AhR(−) shRNA is a sequence which decreases the expression of AhR mRNA by RNA interference. Specimens in italic were analyzed only by qPCR

## Results

### Effects of AhR silencing on BNF-induced mRNA expression of target genes in undifferentiated and differentiated HepaRG cells

**Fig. 1:**
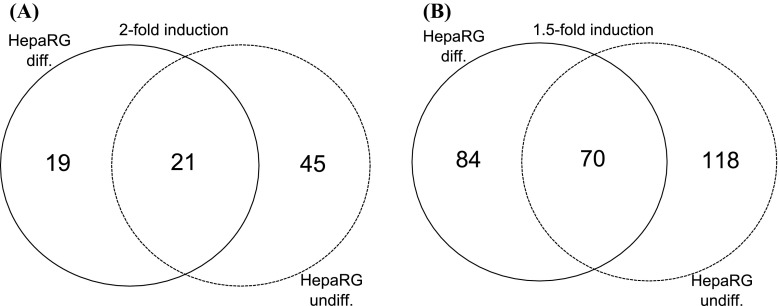
Venn diagram representation of AhR-dependent, BNF-induced genes in differentiated (diff.) and undifferentiated (undiff.) HepaRG cells. Diagram **a** presents number of genes induced at least twofold (*P* ≤ 0.05), whereas diagram **b** represents genes induced at least 1.5-fold (*P* ≤ 0.05) by BNF treatment. Genes were induced in AhR-dependent manner as AhR silencing significantly reduced expression of discussed genes (*P* ≤ 0.05). Detailed list of genes presented in Supplementary 2

AhR-dependent induction of expression of numerous genes was observed after BNF treatment of HepaRG cells. A list of 20 most inducible genes by BNF treatment, as determined by microarray-based gene expression analysis, in HepaRG undifferentiated and differentiated cells is presented in Tables 3 and 4, respectively. Only AhR-dependent effects of BNF treatment were further analyzed in presented publication, i.e., such effects which were significantly suppressed after reduction of *AhR* expression by RNA interference. Quantitative PCR analysis revealed that *AhR* mRNA expression was reduced after transfection of the silencing vector by about 77 and 89% in undifferentiated and differentiated HepaRG cells, respectively (Table 8). Similar results can be calculated from microarrays (see supplementary material). It is always a matter of investigator’s arbitrary choice, how strong effect should be to balance the specificity and the sensitivity of the analysis. Statistically significant induction of expression of 66 genes in undifferentiated cells and induction of expression of 40 genes in differentiated cells was observed after BNF treatment, when twofold effect was chosen as cutoff point (full list of induced genes in supplementary material). The resulting gene sets were compared using Venn diagram analysis in order to examine overlaps among the different gene sets, indicating on 21 genes mutually induced twofold or more in undifferentiated as well as differentiated HepaRG cells (Fig. 1). However, such stringent criterion eliminated most of genes involved in metabolism of xenobiotics and previously recognized as AhR-dependent. The expression of only two genes from classical AhR-dependent battery of genes, namely genes encoding cytochromes P450-*CYP1A1* and *CYP1B1*, were induced more than twofold by BNF treatment. When cutoff point was reduced to 1.5-fold, 188 and 154 genes were upregulated in undifferentiated and differentiated HepaRG cells, respectively. Only 70 genes coexist on both lists (Fig. 1 and supplementary). However, most of genes from AhR-dependent battery of genes are presented on the list this time (supplementary material). Further reduction of cutoff point, below 1.5-fold effect, resulted in substantial increase of casual results, and so was generally omitted in data analysis. It is worth to notice that the expression of another gene from cytochrome P450 family, namely *CYP19A1* encoding aromatase, was induced more than 1.5-fold by BNF treatment, but only in undifferentiated HepaRG cells (supplementary material). The induction was AhR-dependent. Nonetheless, the expression of *CYP19A1* was slightly or not induced after BNF treatment of differentiated HepaRG cells. But alike as in undifferentiated cells, the expression of *CYP19A1* mRNA in differentiated ones was significantly reduced after silencing of AhR (see supplementary material). Functional analysis of genes selected by Venn diagram was performed using DAVID online tools. From 70 genes induced by BNF treatment in AhR-dependent way in both—differentiated and undifferentiated HepaRG cells—as much as ten appear to have connection with regulation of apoptosis and seven is involved in cell proliferation. It is worth to mention that the expression of as much as five genes from solute carrier family of transporters (SLC) was induced by BNF treatment in AhR-dependent manner. The remaining genes are involved in numerous different biological pathways, without obvious domination of one of them.

**Table 3:** Top 20 most inducible by BNF treatment and AhR-dependent genes as determined by microarray-based gene expression analysis in HepaRG undifferentiated cells

Gene (symbol)	Accession No.	BNF induction (contr.BNF/contr. DMSO)		AhR knockdown (AhR(−) BNF/contr. BNF)	
		Fold-change	*P* value	Fold-change	*P* value
CYP1A1	NM_000499	84.4479	1.96E-09	−2.26041	4.43E-05
SERPINB2	NM_002575	38.9277	5.37E-10	−1.74217	3.88E-05
TMEM156	NM_024943	17.0552	1.31E-06	−4.25365	6.58E-05
CYP1B1	NM_000104	9.99312	6.71E-06	−4.8724	0.000114
TIPARP	NM_015508	9.97469	6.29E-07	−3.38236	2.62E-05
TAC1	NM_003182	7.52859	2.97E-06	−5.1163	1.03E-05
SLC7A11	NM_014331	6.84783	9.66E-07	−1.89915	0.000527
SLC7A5	NM_003486	6.65738	3.53E-06	−5.4473	6.80E-06
IGFBP1	NM_000596	6.23676	7.86E-08	−1.89982	3.77E-05
SCG5	NM_003020	5.92127	5.02E-06	−5.22126	7.71E-06
SLC37A2	NM_198277	5.91685	2.40E-06	−2.79372	5.83E-05
AMIGO2	NM_181847	5.36008	1.15E-05	−3.69017	4.90E-05
SLC14A1	NM_015865	5.29511	1.02E-05	−17.1535	4.38E-07
EREG	NM_001432	4.41706	2.30E-05	−1.89436	0.002332
ARL4C	NM_005737	3.87399	3.59E-07	−1.30965	0.003173
PXK	NM_017771	3.8716	1.52E-07	−2.37851	2.14E-06
HMGA2	NM_003484	3.81293	1.82E-07	−1.44529	0.000326
HK2	NM_000189	3.63351	4.47E-05	−2.41403	0.000378
STC2	NM_003714	3.52418	8.44E-07	−1.67544	0.00015
KYNU	NM_003937	3.39497	2.82E-06	−1.89998	0.000117

Information of all additional genes out of top 20 is available in the supplementary material accompanying of the manuscript (Supplementary 2—induction)

Fold-change value that was less than 1 has been replaced by the negative of its inverse (for example, 0.1 was replaced by −10)

*Contr.* HepaRG cells transfected with control plasmid, *AhR* ^*−*^ HepaRG cells transfected with plasmid knocking down Ah receptor

**Table 4:** Top 20 most inducible by BNF treatment and AhR-dependent genes as determined by microarray-based gene expression analysis in HepaRG differentiated cells

Gene (symbol)	Accession No.	BNF induction (contr.BNF/contr. DMSO)		AhR knockdown (AhR(−) BNF/contr. BNF)	
		Fold-change	*P* value	Fold-change	*P* value
SERPINB2	NM_002575	19.263	1.93E-09	−11.1499	6.56E-09
CYP1A1	NM_000499	10.9253	7.89E-08	−2.76109	1.25E-05
STC2	NM_003714	7.26696	3.70E-07	−1.98847	0.000175
ARL4C	NM_005737	6.70831	4.72E-08	−3.32309	7.33E-07
TIPARP	NM_015508	6.0173	2.73E-06	−6.40034	2.24E-06
SCG5	NM_003020	5.95549	4.92E-06	−14.4314	4.56E-07
CYP1B1	NM_000104	4.76405	6.34E-05	−9.085	8.60E-06
SLC37A2	NM_198277	4.33837	4.18E-07	−4.1171	5.19E-07
SLC7A5	NM_003486	3.50711	3.89E-05	−3.25045	5.57E-05
BMPER	NM_133468	3.4992	4.40E-05	−5.24619	8.70E-06
SYNJ2	NM_003898	3.41386	1.11E-06	−2.14635	1.79E-05
SLC14A1	NM_015865	2.61341	0.000938	−19.6687	1.51E-06
GDF15	NM_004864	2.5802	0.000948	−3.18849	0.000321
KIFC3	NM_005550	2.5573	8.61E-06	−1.62367	0.000369
UGCG	NM_003358	2.55685	1.05E-05	−2.60471	9.33E-06
ATF3	NM_004024	2.52517	3.11E-05	−1.33103	0.013789
PXK	NM_017771	2.51771	7.21E-07	−3.20378	1.82E-07
MYADM	NM_138373	2.5164	2.96E-05	−1.36498	0.009073
SSH1	NM_018984	2.49408	0.000607	−2.37291	0.000818
IL8	NM_000584	2.47773	1.87E-05	−1.80874	0.000211

Information of all additional genes out of top 20 is available in the supplementary material accompanying of the manuscript (Supplementary 2—induction)

Fold-change value that was less than 1 has been replaced by the negative of its inverse (for example, 0.1 was replaced by −10)

*Contr.* HepaRG cells transfected with control plasmid, *AhR* ^*−*^ HepaRG cells transfected with plasmid knocking down Ah receptor

### Effects of *AhR* silencing on BNF-inhibited mRNA expression of target genes in undifferentiated and differentiated HepaRG cells

**Fig. 2:**
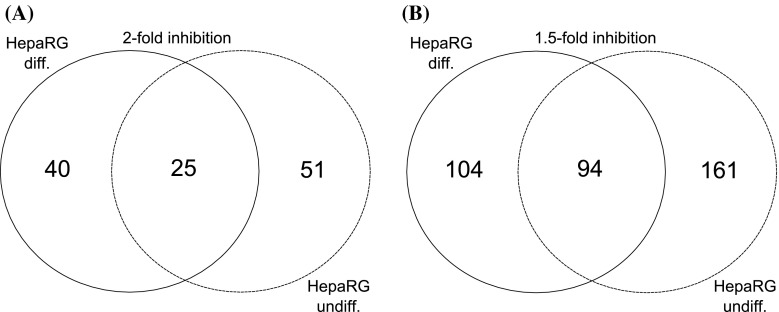
Venn diagram representation of AhR-dependent, BNF-inhibited genes in differentiated (diff.) and undifferentiated (undiff.) HepaRG cells. Diagram **a** presents number of genes inhibited at least twofold (*P* ≤ 0.05), whereas diagram **b** represents genes inhibited at least 1.5-fold (*P* ≤ 0.05) by BNF treatment. Genes were inhibited in AhR-dependent manner as AhR silencing significantly increased expression of discussed genes (*P* ≤ 0.05). Detailed list of genes presented in Supplementary 3

A list of 20 most inhibited genes by BNF treatment, as determined by microarray-based gene expression analysis, in HepaRG undifferentiated and differentiated cells is presented in Tables 5 and 6, respectively. Statistically significant reduction of expression of 76 genes in undifferentiated cells and reduction of expression of 65 genes in differentiated cells was observed after BNF treatment, when twofold effect was chosen as cutoff point (Fig. 2, full list of inhibited genes in supplementary material). Expression of 25 genes was reduced simultaneously in undifferentiated as well as in differentiated cells. When cutoff point was reduced to 1.5-fold, 255 and 198 genes were downregulated after BNF treatment in undifferentiated and differentiated HepaRG cells, respectively. Expression of 94 of them was reduced concomitantly in both stages of HepaRG differentiation (Fig. 2 and supplementary). Interestingly, the expression of *GSTA1* and *GSTA2* was downregulated after BNF treatment in AhR-dependent manner (Table 6). Functional analysis of 94 genes selected by Venn diagram revealed that ten of them appeared to be connected with cell adhesion, five of them are engaged in formation of anchoring junction, and another five are connected with response to steroid hormone stimulus. The remaining genes are dispersed between numerous different biological pathways.

**Table 5:** Top 20 most inhibited by BNF treatment and AhR-dependent genes as determined by microarray-based gene expression analysis in HepaRG undifferentiated cells

Gene (symbol)	Accession No.	BNF repression (contr.BNF/contr. DMSO)		AhR knockdown (AhR(−) BNF/contr. BNF)	
		Fold-change	*P* value	Fold-change	*P* value
KIAA1456	NM_020844	−4.45339	9.35E-05	2.06645	4.25E-03
KDR	NM_002253	−4.13891	4.18E-06	1.5346	3.18E-03
FGG	NM_021870	−3.98685	9.06E-07	2.38767	1.38E-05
ART3	NM_001179	−3.83642	1.48E-06	9.44067	7.00E-08
KCNB1	NM_004975	−3.65479	1.44E-05	3.2784	2.39E-05
FAM65B	NM_014722	−3.559	1.79E-04	2.76605	5.99E-04
PLCL1	NM_006226	−3.51205	7.21E-05	1.60191	1.12E-02
MLIP	NM_138569	−3.50818	3.56E-07	3.30055	4.79E-07
PPL	NM_002705	−3.47763	4.52E-05	4.68824	1.31E-05
MCF2	NM_005369	−3.44442	1.67E-05	1.81629	9.73E-04
CIDEC	NM_022094	−3.39535	5.04E-06	2.71645	1.63E-05
PDE1A	NM_005019	−3.35762	5.66E-05	3.50297	4.65E-05
CIDEC	NM_022094	−3.32232	2.55E-05	2.76084	6.65E-05
PLCL1	NM_006226	−3.24423	8.63E-05	1.54723	0.013509
SAA2	NM_030754	−3.19599	0.003267	3.18935	0.003296
NRXN3	NM_004796	−3.15472	1.60E-05	4.53509	3.21E-06
MUM1L1	NM_152423	−3.14915	6.06E-06	1.81139	0.000261
FLRT3	NM_013281	−3.14526	2.78E-06	2.13495	3.09E-05
SORBS1	NM_006434	−3.11927	0.000186	2.49061	0.000621
FABP4	NM_001442	−3.0878	1.80E-05	25.5207	3.50E-08

Information of all additional genes out of top 20 is available in the supplementary material accompanying of the manuscript (Supplementary 3—inhibition)

Fold-change value that was less than 1 has been replaced by the negative of its inverse (for example, 0.1 was replaced by −10)

*Contr.* HepaRG cells transfected with control plasmid, *AhR* ^*−*^ HepaRG cells transfected with plasmid knocking down Ah receptor

**Table 6:** Top 20 most inhibited by BNF treatment and AhR-dependent genes as determined by microarray-based gene expression analysis in HepaRG differentiated cells

Gene (symbol)	Accession No.	BNF repression (contr.BNF/contr. DMSO)		AhR knock down (AhR(−) BNF/contr. BNF)	
		Fold-change	*P* value	Fold-change	*P* value
CYP4F3	NM_000896	−3.44897	6.68E-05	1.91091	0.002218
ABCD2	NM_005164	−3.3931	2.81E-06	3.16358	3.98E-06
GSTA2	NM_000846	−3.2792	0.00027	2.57352	0.000923
PPL	NM_002705	−3.15243	7.22E-05	5.03675	1.00E-05
LIFR	NM_002310	−3.12767	0.000326	2.07572	0.003313
GSTA1	NM_145740	−3.1041	6.89E-06	1.97077	0.000131
SLC38A4	NM_018018	−3.01334	3.39E-05	1.53637	0.005241
SPP1	NM_000582	−2.98976	2.13E-05	1.7532	0.000884
PDZK1	NM_002614	−2.90891	4.81E-06	1.36698	0.004118
FAM65B	NM_014722	−2.85695	0.000506	5.59945	3.16E-05
MCF2	NM_005369	−2.74897	1.15E-05	3.97328	1.85E-06
CTGF	NM_001901	−2.64531	9.64E-06	3.91883	1.31E-06
PLAC8	NM_016619	−2.59761	5.42E-05	1.49609	0.005266
CALCR	NM_001742	−2.59678	1.42E-05	1.38406	0.004838
AKR1B10	NM_020299	−2.59623	5.78E-05	21.371	6.06E-08
CYP3A4	NM_017460	−2.58788	0.000534	1.83438	0.005209
SEMA3C	NM_006379	−2.57786	0.001181	1.74833	0.014406
RDH5	NM_002905	−2.54186	1.26E-05	12.5243	3.45E-08
PKP2	NM_004572	−2.53416	0.000395	1.63935	0.009291
KCNB1	NM_004975	−2.50343	0.000104	3.24028	2.54E-05

Information of all additional genes out of top 20 is available in the supplementary material accompanying of the manuscript (Supplementary 3—inhibition)

Fold-change value that was less than 1 has been replaced by the negative of its inverse (for example, 0.1 was replaced by −10)

*Contr.* HepaRG cells transfected with control plasmid, *AhR(−)* HepaRG cells transfected with plasmid knocking down Ah receptor

### Diverse, dependent on the stage of cell differentiation, effects of AhR silencing and BNF treatment on mRNA expression of some target genes

If Ah receptor is responsible for BNF-related induction of appropriate genes, someone could expect that silencing of AhR would reduce such induction. Indeed, expression of most of the analyzed genes followed this pattern (Table 3). However, expression of several genes seems not to follow such simplified rules. Two examples of such genes are presented in Table 7. Expression of the first one, cyclin E2 (*CCNE2*), was significantly induced by BNF treatment of undifferentiated HepaRG cells. However, silencing of Ah receptor not only did not counteract such induction, but also further increased the expression of AhR-silenced, BNF-treated cells as compared to control BNF-treated undifferentiated HepaRG cells. This paradoxical effect was statistically significant and indicates the involvement of AhR. Distinct to above, but consistent with expectation, pattern of *CCNE2* expression was observed in differentiated HepaRG cells. BNF treatment slightly induced *CCNE2* mRNA expression and AhR silencing significantly reduced this induction this time as well (Table 7). Two distinct, complementary to *CCNE2* mRNA probe sets are placed on Affymetrix U219 array chip. Each one consists of eleven 25 base oligomers spanning the region of 818–1299 bp (set 11728301_at) and 2137-2636 bp (set 11728300_at) of the reference mRNA sequence (NM_057749). The results of cDNA hybridization to both probe sets were consistent to one another (Table 7) and are also supported by qPCR expression analysis (Table 8). DNA region amplified by quantitative real-time PCR was localized between 888 and 1001 base of NM_057749 sequence. Likewise, the expression of the second depicted gene, interleukin 8 (*IL8*), followed very similar pattern to *CCNE2* one. The results of cDNA hybridization to all three *IL8* probe sets were consistent to one another and indicated on differences between differentiated and undifferentiated HepaRG cells (Table 7).

**Table 7:** Cell differentiation-dependent effects of AhR silencing and BNF treatment on mRNA expression of *CCNE2* and *IL8* genes

Cell types	Probe set ID.	BNF induction (contr.BNF/contr. DMSO)		AhR knockdown (AhR(−)BNF/contr. BNF)	
		Fold-change	*P* value	Fold-change	*P* value
CCNE2 (NM_057749)					
HepaRG undifferentiated	11728300_at	4.55583	1.77E-06	2.38103	4.59E-05
	11728301_at	2.60968	2.54E-05	2.08231	0.000117
HepaRG differentiated	11728300_at	1.37344	0.008872	−1.57768	0.001553
	11728301_at	1.11149	0.250375	−1.30069	0.019462
IL8 (NM_000584)					
HepaRG undifferentiated	11718841_s_at	3.3702	3.39E-06	1.35246	0.006721
	11754026_a_at	5.29994	0.000192	1.60235	0.062341
	11763226_x_at	3.64619	1.85E-05	1.56707	0.005473
HepaRG differentiated	11718841_s_at	2.47773	1.87E-05	−1.80874	0.000211
	11754026_a_at	2.30724	0.006706	−1.7321	0.037385
	11763226_x_at	1.6419	0.003416	−1.53201	0.006949

**Table 8:** Validation of selected microarray-based genes expression data by real-time quantitative PCR

	Relative expression of mRNA ± SD							
Type of cells	HepaRG undifferentiated				HepaRG differentiated			
Transfected vector	Negative control		AhR(−)		Negative control		AhR(−)	
Treatment	Solvent	BNF	Solvent	BNF	Solvent	BNF	Solvent	BNF
Gene								
CYP1A1	0.405 ± 0.2113	100.0 ± 57.28^a^	4.091 ± 2.582	49.51 ± 42.20	3.132 ± 1.569	32.22 ± 14.21^a^	8.803 ± 6.778	14.67 ± 12.42
GSTA2	70.44 ± 3.862	17.62 ± 5.749^a^	34.94 ± 3.833	13.91 ± 4.109^a^	52.89 ± 33.33	13.75 ± 5.733	100.0 ± 80.27	30.93 ± 13.82
SERPINB2	1.205 ± 0.6849	100.0 ± 10.22^a^	1.234 ± 1.032	20.73 ± 13.18^b^	1.173 ± 0.1412	33.80 ± 11.94^a^	0.2250 ± 0.1469	2.713 ± 1.336^b^
SLC7A5	6.274 ± 1.461	100.0 ± 22.94^a^	2.963 ± 0.5229	18.74 ± 10.17^b^	2.547 ± 0.6880	89.42 ± 28.47^a^	1.687 ± 0.5035^c^	7.641 ± 2.617^ab^
SLC14A1	11.15 ± 0.1337	100.0 ± 34.49^a^	2.307 ± 1.731	3.675 ± 3.376^b^	52.47 ± 36.10	78.07 ± 19.72	4.562 ± 3.840	2.147 ± 0.7728^b^
CCNE2	18.77 ± 9.006	53.92 ± 16.49^a^	24.98 ± 11.91	100.0 ± 14.59^ab^	52.82 ± 6.010	71.14 ± 11.51	35.74 ± 8.194	32.58 ± 17.95^b^
TIPARP	13.22 ± 2.026	98.99 ± 10.46^a^	11.67 ± 1.943	18.42 ± 8.759^b^	24.62 ± 2.026	100.0 ± 24.18^a^	24.45 ± 3.926	24.22 ± 4.037^b^
STC2	35.39 ± 16.29	100.0 ± 22.33^a^	43.16 ± 21.43	62.09 ± 21.18	18.25 ± 12.41	92.41 ± 78.18	17.65 ± 3.669	57.64 ± 49.78
SCG5	12.95 ± 8.600	100.0 ± 47.09^a^	1.514 ± 0.6559	17.69 ± 15.42^b^	2.632 ± 1.659	9.453 ± 5.483	0.1560 ± 0.1291	0.9109 ± 0.6250^b^
TMEM156	12.75 ± 7.720	100.0 ± 22.84^a^	4.393 ± 1.505	21.13 ± 11.91^b^	1.233 ± 0.0565	7.672 ± 0.4095^a^	0.4391 ± 0.1383^c^	1.158 ± 0.2322^ab^
AhR	37.51 ± 9.057	33.92 ± 4.433	7.732 ± 1.840^c^	9.052 ± 3.721^c^	100.0 ± 23.76	81.13 ± 10.21	11.11 ± 4.527^c^	9.161 ± 0.9119^c^

^a^Significantly different from solvent-treated cells

^b^Significantly different from BNF-treated negative control transfected cells

^c^Significantly different from negative control transfected cells

### Real-time PCR validation of microarray-based genes expression data

The results of our qPCR experiments are presented as relative expression of the genes (Table 8). Expression of *AhR* mRNA was determined to demonstrate real effectiveness of our *AhR* silencing construct on mRNA level. On the other hand, expression of *CYP1A1* mRNA, model gene regulated by Ah receptor, showed how changes of *AhR* mRNA translate to the receptor function. Genes such as *SERPINB2*, *SLC7A5*, *SLC14A1*, *CCNE2*, *TIPARP*, *STC2*, *SCG5,* and *TMEM156*, which expression was validated by real-time PCR, belonged to the most inducible by BNF genes but outside classical AhR-dependent genes battery, or as in the case of *GSTA2*, regulated in opposite direction as was expected. As it was written in the previous chapter, the expression of *CCNE2* was determined by real-time PCR because microarray analysis suggested very strange and unexpected regulation of this gene expression by Ah receptor.

Comparison of two different treatments groups, both with very low gene expression, could result in multiplying stochastic errors. Thus, the knowledge of approximate level of particular gene expression appeared to be a valuable one. We did not determine efficiencies of reverse transcription of particular mRNAs; therefore, we could not present our results as “absolute” quantification, e.g., as exact mRNA copy number. However, to determine the limit of detection and the efficiency of PCR amplification, we have used calibration (dilution) curve from which we could anticipate the approximate copy number of particular cDNAs in our PCR reaction (see “Materials and methods” section). Thus, the values of 100.0 presented in Table 8 correspond to 2.34 × 10^3^ molecules of CYP1A1 cDNA in 0.4 µl of undiluted reverse-transcription products, 159 molecules for GSTA2, 4.31 × 10^3^ molecules for SERPINB2, 209 molecules for SLC7A5, 76 molecules for SLC14A1, 916 molecules for CCNE2, 2.63 × 10^3^ molecules for TIPARP, 589 molecules for STC2, 4.57 × 10^3^ molecules for SCG5, 1.45 × 10^3^ molecules for TMEM156, and 19.9 × 10^3^ molecules for AhR.

## Discussion

The aryl hydrocarbon receptor is a ligand-activated transcription factor involved in many physiological processes. In laboratory animals, genetic variations in the *AhR* lead to significant differences in sensitivity to biochemical and carcinogenic effects of PAHs, TCDD, and related compounds [50]. Since late fifties till the end of twentieth century, most aspects of AhR function were contributed to its ability to induce enzymes responsible for metabolism of xenobiotic, drugs, and carcinogens [1, 51]. The situation has changed together with the dawn of microarray era at the beginning of twentieth century . It was demonstrated by gene expression profiling studies that AhR is responsible for induction or repression of hundreds of other genes, supposedly not directly connected to metabolism of xenobiotics [21, 27, 31, 52–57]. Generally, our results confirm the above observations. From 21 genes induced more than twofold by BNF treatment in both, undifferentiated and differentiated HepaRG cells, only cytochromes *CYP1A1* and *CYP1B1* belonged to classical AhR-dependent battery of genes encoding enzymes involved in metabolism of xenobiotics. However, when stringency of cutoff criterion was reduced to 1.5-fold, AhR-specific induction of *ALDH3A1*, *NQO1*, and *UGT1A1* expression by BNF treatment has been observed. However, to our surprise, we did not observe AhR-dependent induction of *CYP1A2* expression after BNF treatment of the cells. Induction of *CYP1A2* expression after treatment of animals or human cell lines with diverse AhR ligands was widely demonstrated in many publications in this field. It was observed also after treatment of HepaRG with either TCDD [40] or BNF [41]. Our earlier experiments with BNF treatment of HepaRG cells were performed in virtually identical conditions as performed herein [41], except one substantial difference—in our earlier work, we had used unmodified HepaRG cells, whereas in the present study, HepaRG cell line was stably transfected with either control or Ah(−) pGeneClip™ vectors. It is possible that transfected control vector interfered somehow with expression of *CYP1A2* mRNA, either by accidental localization of the vector integration site nearby the gene’s locus or by interference of negative control shRNA with the gene’s RNA. However, the second case is unlikely, as negative control shRNA is a scrambled artificial sequence which does not match any human gene. Another possibility which cannot be excluded is some kind of interference between the expression of *CYP1A2* and RNA transcribed from neomycin or ampicillin resistance genes present on shRNA plasmids.

Our results suggest the involvement of Ah receptor in the regulation of *CYP19A1*, another member of the cytochrome P450 superfamily as well. Protein product of *CYP19A1* known as aromatase is an enzyme responsible for a key step in the biosynthesis of estrogens. Cross-talk of Ah receptor and estrogen receptor 1 (ER) signaling pathways are well described, but the underlying molecular mechanisms have been largely elusive. Interactions between these two pathways have been proposed to be due to a combination of several different mechanisms including increased metabolism of estrogen mediated by the AhR-dependent expression of *CYP1A1* and *CYP1B1* [58], direct interaction between AhR and ER [59], synthesis of inhibitory factors [60], direct inhibition through inhibitory XREs located in estrogen-responsive gene promoters [61], and increased ER degradation [62]. AhR-dependent induction of *CYP19A1* expression by BNF treatment of HepaRG cells could be considered as another mechanism of AhR and ER pathways intersection. Our results are consistent with earlier findings describing AhR-dependent regulation of *CYP19A1* expression in mouse ovarian granulosa cells [63]. BNF-related induction of *CYP19A1* expression could explain some estrogen-like effects of BNF treatment of ovariectomized rats as well [22]. However, hepatocytes surely are not a primary source of the aromatase activity.

The reactive metabolites formed from xenobiotics by cytochromes P450 are usually detoxified to more polar products by phase II conjugative enzymes, such as GSTA1 [5]. Rodent *Gsta1(GstYa)* is known to be a target gene of AhR [2, 64]. It was demonstrated that the expression of *GstYa* was induced in the liver after BNF treatment of rats [65]. Human *GSTA1* and its paralog *GSTA2* are the orthologs of rodent *GstYa* gene. Consequently, it should be expected that BNF treatment of human HepaRG cells would increase the expression of *GSTA* genes as well. Our previous results suggested that the expression of *GSTA1* was regulated by AhR in unmodified HepaRG cells [41]. Present results did confirm this suggestion. Indeed, expression of both, *GSTA1* and *GSTA2*, was regulated by AhR. However, instead of anticipated induction, we have noticed significant inhibition of *GSTA1* and *GSTA2* expression following BNF treatment of HepaRG cells and this effect was significantly reduced after AhR knockdown by means of RNA interference. In our previous work, we hypothesized that maybe the decrease of *GSTA1* expression after BNF treatment is compensated by simultaneous induction of some other GST isoenzymes [41]. Our present results did not confirm the above hypothesis. Analysis of the expression of genes by microarrays indicated that none of the GST isoenzymes were induced by BNF treatment of HepaRG cells, at least in investigated time point. Additional studies, especially at different time points, are necessary to determine if AhR-dependent inhibition of *GSTA1* and *GSTA2* by BNF treatment of HepaRG cells depicts interspecies differences between human and rodents, is model specific confined only to HepaRG cell line, or maybe is a result of different timing's or ligand's specificity.

Differentiated and undifferentiated HepaRG cells are genetically identical but committed to diverse gene expression programs. Consequently, our results clearly demonstrate different gene expression profiles between differentiated and undifferentiated cells. Barely about 25% of AhR-dependent genes were mutually induced and roughly 26% of genes were mutually inhibited in undifferentiated as well as in differentiated HepaRG cells. Therefore, levels of cell differentiation followed by condition of cell culture appeared to be much more important than the genetic background for pattern of activity of AhR-dependent genes. The above-mentioned conclusion is consistent with our earlier findings where expression of some AhR-dependent genes was compared between both, differentiated and undifferentiated, unmodified HepaRG cells [41]. The conclusion is consistent also with the findings of involvement of AhR in development of fetal mouse liver [42] or control of expansion of human hematopoietic stem cells in culture [66]. It was demonstrated that different cell types were involved in AhR-dependent development of mouse liver and in AhR-dependent hepatotoxicity [43]. Taken together, as undifferentiated HepaRG cells could be considered as similar to cells from fetal liver or stem cells, whereas differentiated ones resemble maturated hepatocytes, so both variants of cell differentiation stages generate distinct pattern of expression of AhR-dependent genes.

Likewise, analysis of effects of BNF treatment and *AhR* silencing on the expression of genes such as interleukin 8 (*IL8*) or cyclin E2 (*CCNE2*), indicated on predominant influence of cell differentiation stages in AhR-dependent regulation of the gene expression. Expression of *IL8* has been already reported as AhR-dependent [67–69]. However, induction of *IL8* expression by AhR ligands is supposed to be mediated by different from classical mechanism. Instead ARNT, liganded AhR binds to RelB and such heterodimer activates ReIB/AhR-responsive element of the *IL*-*8* promoter. Postulated ReIB/AhR-responsive element differs from classical XRE [69]. To our best knowledge, *CCNE2* expression has been not connected to AhR yet. However, analysis of the promoter of *CCNE2* indicated on 2 classical core XRE sequences localized -824 and -559 bp upstream to the transcription starting site. On the contrary to *IL8*, we did not found any RelB/AhR-responsive element in the promoter of *CCNE2*. Nevertheless, treatment with BNF significantly induced expression of both genes in undifferentiated HepaRG cells, but silencing of Ah receptor not only did not counteract of such induction, but also further increased the expression of both genes in AhR-silenced, BNF-treated cells as compared to control BNF-treated undifferentiated HepaRG cells. This paradoxical effect was statistically significant and indicated on involvement of AhR. It was observed only in undifferentiated HepaRG cells. As far as differentiated HepaRG cells concerned, AhR-dependent response of *IL8* and *CCNE2* expression to BNF treatment proceeded according to the expectations of investigators. In this case, BNF treatment of differentiated HepaRG cells resulted in significant induction of *IL8* and *CCNE2* expression, respectively, and the induction was significantly reduced after knocking down AhR. Attempts to explain above phenomenon are difficult and can be only speculative at present state of our knowledge. Differentiated HepaRG cells are committed to another gene expression program with different patterns of transcriptionally active chromatin than undifferentiated ones. Maybe some differentiation-dependent modifications of *CCNE2* and *IL8* gene promoters’ structure followed by diverse accessibility for transcription factors cooperating with AhR could explain discussed results. Additional studies are necessary to explain the observed phenomenon.

Direct comparison of our results with different microarray studies is difficult, as most of other studies used TCDD as an AhR ligand and substantial differences between diverse AhR ligands, including BNF and TCDD, were observed [31]. Likewise, substantial differences between different species [34], rat strains [56], and different mouse tissues [27] were reported. Similar to above, substantial differences in respect to expression of AhR-dependent genes encoding xenobiotic-metabolizing enzymes were observed in undifferentiated as compared to differentiated HepaRG cells after BNF treatment [41]. Nevertheless, some well-established, AhR ligands regulating genes were induced despite of different species, strains, ligands, and tissues. Apart from cytochromes P450, *SERPINB2* and *TIPARP* belonged to the most inducible by BNF- and AhR-dependent genes in both undifferentiated and differentiated HepaRG lines. *SERPINB2* was reported in different human cell lines as inducible by TCDD treatment [70–73]. We demonstrated that expression of *SERPINB2* was induced by BNF treatment of HepaRG cells as well. As a matter of fact, *SERPINB2* was the most inducible gene in differentiated and the second one after *CYP1A1* in undifferentiated HepaRG cells, respectively. It is especially interesting as the precise role of *SERPINB2* remains an enigma [74, 75] and its connection to cancer has been reported [76]. The expression of *TIPARP* was also reported to be regulated by TCDD via activation of the AhR [77, 78]. Our results demonstrate that BNF is also a potent inducer of *TIPARP* expression. Very efficient induction of *TIPARP* expression by BNF in HepaRG cells is somehow contradictory to identification of *TIPARP* as the gene that can mediate TCDD toxicity by suppression of hepatic gluconeogenesis [79]. In short-term toxicity studies in animals, the typical effects of exposition on TCDD were wasting syndrome and thymus atrophy [80]. To our knowledge, such effects were never observed after exposition of animals to BNF, even after the 9-dose treatment of rats with BNF, the treatment which according to intention of investigators was supposed to mimic the effect of exposition to persistent TCDD [22]. If elevated, the expression of *TIPARP* would be accountable for TCDD-mediated toxicity, it should be expected that BNF is not effective inducer of this gene. However, our results did not confirm the above expectation, at least in HepaRG cell line. In addition, it was reported that TIPARP is a repressor of AhR transactivation, revealing a new mechanism of negative feedback control in AhR signaling [81]. Such negative feedback control of *AhR* expression by *TIPARP* in HepaRG cell line could be of particular importance, as expression of *AHRR*, the other negative controller of AhR [82], appears to be on a very low level as observed herein and in our earlier study [41].

Functional analysis of genes induced or inhibited by BNF treatment of HepaRG cells revealed involvement of these genes in multiple biological pathways, not directly connected to metabolism of xenobiotics. As a matter of fact, genes involved in metabolism of xenobiotics constitute only minute fraction of all genes regulated by AhR. Participation of the aryl hydrocarbon receptor in induction of expression of genes connected to regulation of apoptosis or involved in cell proliferation from one side, and in inhibition of genes connected to cell adhesion from the other side could explain some results suggesting involvement of AhR not only in initiation but also in progression of cancer [83]. In agreement with above, novel physiological function for AhR has been proposed recently, as regulator of self-renewal of hematopoietic stem cells [66] or in general, modulator of the balance between differentiation and pluripotentiality in normal and transformed tumor cells [84].

Current work in our laboratory aims to identify possible function of some AhR-dependent genes selected in this study.

## Electronic supplementary material

Below is the link to the electronic supplementary material.

Supplementary material 1 (XLSX 32272 kb)

Supplementary material 2 (DOCX 32 kb)

Supplementary material 3 (DOCX 22 kb)

## Abbreviations

AhR: Aryl hydrocarbon receptor
ARNT: AhR nuclear translocator
AhRR: AhR repressor
CYP1A1: Cytochrome P450 1 family, member A1
CYP1A2: Cytochrome P450 1 family, member A2
CYP1B1: Cytochrome P450 1 family, member B1
ER: Estrogen receptor
STC2: Stanniocalcin 2
ARL4C: ADP-ribosylation factor-like 4C
TIPARP: TCDD-inducible poly(ADP-ribose) polymerase
CCNE2: Cyclin E2
IL8: Interleukin 8
NQO1: NAD(P)H quinone oxidoreductase 1
GSTA2: Glutathione transferase A2
SLC: Solute carrier family
SCG5: Secretogranin V (7B2 protein)
TMEM156: Transmembrane protein 156
ALDH3A1: Aldehyde dehydrogenase 3 family, member A1
UGT1A1: UDP-glucuronosyltransferase 1 family, member A1
BNF: β-Naphthoflavone
3-MC: 3-methylcholanthrene
TCDD: 2,3,7,8-tetrachlorodibenzo-*p*-dioxin
PAH: Polycyclic aromatic hydrocarbon
