# Supplementary material for: Induction of expression of aryl hydrocarbon receptor-dependent genes in human HepaRG cell line modified by shRNA and treated with β-naphthoflavone
Source: Mol Cell Biochem. 2016 Oct 28;425(1):59–75. doi: 10.1007/s11010-016-2862-3 (PMC5225230; doi:10.1007/s11010-016-2862-3)
Supplement: Supplementary file 2 — Supplementary material 2 (DOCX 32 kb) [file 11010_2016_2862_MOESM2_ESM.docx]

List of genes induced in AhR dependent manner at least 1.5-fold (P≤0.05) by BNF treatment of undifferentiated and differentiated HepaRG cells. Data presented in Fig.1.

1. **Genes induced in both, undifferentiated as well as differentiated HepaRG cells (70 genes).**

NM_000104 - CYP1B1, cytochrome P450, family 1, subfamily B, polypeptide 1;

NM_001144757 /// NM_003020 - SCG5, secretogranin V (7B2 protein);

NM_005475 - SH2B3, SH2B adaptor protein 3;

NM_003486 - SLC7A5, solute carrier family 7 (cationic amino acid transporter, y+ system), member 5;

NM_015221 – DNMBP, dynamin binding protein;

NM_000676 - ADORA2B, adenosine A2b receptor;

NM_022842 /// NM_178181 - CDCP1, CUB domain containing protein 1;

NM_001100818 /// NM_017933 - PID1, phosphotyrosine interaction domain containing 1;

NM_000729 – CCK, Cholecystokinin;

NM_058238 - WNT7B, wingless-type MMTV integration site family, member 7B;

NM_001009880 /// NM_015264 - C22orf9, chromosome 22 open reading frame 9;

NM_033393 - FHDC1, FH2 domain containing 1;

NM_014550 - CARD10, caspase recruitment domain family, member 10;

NM_002430 - MN1, meningioma (disrupted in balanced translocation) 1;

NM_014331 - SLC7A11, solute carrier family 7, (cationic amino acid transporter, y+ system) member 11;

NM_003714 - STC2, stanniocalcin 2;

NM_033260 - FOXQ1, forkhead box Q1;

NM_000693 - ALDH1A3, aldehyde dehydrogenase 1 family, member A3;

NM_001143818 /// NM_002575 - SERPINB2, serpin peptidase inhibitor, clade B (ovalbumin), member 2;

NM_003358 – UGCG, UDP-glucose ceramide glucosyltransferase;

NM_001130099 /// NM_001130100 /// NM_005550 - KIFC3, kinesin family member C3;

NM_005737 - ARL4C, ADP-ribosylation factor-like 4C;

NM_001032998 /// NM_003937 – KYNU, kynureninase (L-kynurenine hydrolase);

NM_000189 - HK2, hexokinase 2;

NM_014398 - LAMP3, lysosomal-associated membrane protein 3;

NM_133468 – BMPER, BMP binding endothelial regulator;

NM_017771 – PXK, PX domain containing serine/threonine kinase;

NM_024943 - TMEM156, transmembrane protein 156;

NM_005228 /// NM_201282 /// NM_201283 /// NM_201284 – EGFR, epidermal growth factor receptor (erythroblastic leukemia viral (v-erb-b) oncoge;

NM_001145001 /// NM_014397 - NEK6, NIMA (never in mitosis gene a)-related kinase 6;

NM_002627 – PFKP, phosphofructokinase, platelet;

NM_006470 - TRIM16, tripartite motif-containing 16 /// tripartite motif-containing 16-like;

NM_025139 - ARMC9, armadillo repeat containing 9;

NM_001128303 /// NM_153218 - C13orf31, chromosome 13 open reading frame 31;

NM_001113239 /// NM_022740 /// XM_001716827 /// XM_925800 - HIPK2, homeodomain interacting protein kinase 2;

NM_007129 - ZIC2, Zic family member 2 (odd-paired homolog, Drosophila);

NM_001128175 /// NM_001390 /// NM_001391 /// NM_001392 /// NM_032975 /// NM_0329 – DTNA, dystrobrevin, alpha;

NM_021243 - C6orf115, chromosome 6 open reading frame 115;

NM_001042467 /// NM_024101 – MLPH, melanophilin;

NM_001128588 /// NM_001146036 /// NM_001146037 /// NM_015865 - SLC14A1, solute carrier family 14 (urea transporter), member 1 (Kidd blood group);

NM_001145290 /// NM_198277 - SLC37A2, solute carrier family 37 (glycerol-3-phosphate transporter), member 2;

NM_030816 - ANKRD13C, ankyrin repeat domain 13C;

NM_031453 - FAM107B, family with sequence similarity 107, member B;

NM_005779 - LHFPL2, lipoma HMGIC fusion partner-like 2;

NM_001092 /// NM_001159746 /// NM_021962 – ABR, active BCR-related gene;

NM_001128850 /// NM_004165 – RRAD, Ras-related associated with diabetes;

NM_001007075 /// NM_015990 /// NM_199039 - KLHL5, kelch-like 5 (Drosophila);

NM_020760 - HECW2, HECT, C2 and WW domain containing E3 ubiquitin protein ligase 2;

NM_015210 - KIAA0802, KIAA0802;

NM_000499 - CYP1A1, cytochrome P450, family 1, subfamily A, polypeptide 1;

NM_003182 /// NM_013996 /// NM_013997 /// NM_013998 - TAC1, tachykinin, precursor 1;

NM_000610 /// NM_001001389 /// NM_001001390 /// NM_001001391 /// NM_001001392 - CD44, CD44 molecule (Indian blood group);

NM_003059 - SLC22A4, solute carrier family 22 (organic cation/ergothioneine transporter), member 4;

NM_001161330 /// NM_001161331 /// NM_018984 - SSH1, slingshot homolog 1 (Drosophila);

NM_003897 - IER3, immediate early response 3;

NM_001143668 /// NM_181847 - AMIGO2, adhesion molecule with Ig-like domain 2;

NM_007172 /// NM_153645 - NUP50, nucleoporin 50kDa;

NM_001902 /// NM_153742 – CTH, cystathionase (cystathionine gamma-lyase);

NM_002153 - HSD17B2, hydroxysteroid (17-beta) dehydrogenase 2;

NM_002872 - RAC2, ras-related C3 botulinum toxin substrate 2 (rho family, small GTP binding protein;

NM_005559 - LAMA1, laminin, alpha 1;

NM_015508 – TIPARP, TCDD-inducible poly(ADP-ribose) polymerase;

NM_178496 - C3orf59, chromosome 3 open reading frame 59;

NM_004595 – SMS, spermine synthase;

NM_001136053 /// NM_001142646 /// NM_016372 - TPRA1, transmembrane protein, adipocyte asscociated 1;

NM_003898 - SYNJ2, synaptojanin 2;

NM_153360 - APCDD1L, adenomatosis polyposis coli down-regulated 1-like;

NM_033120 - NKD2, naked cuticle homolog 2 (Drosophila);

NM_001015051 /// NM_001024630 /// NM_004348 - RUNX2, runt-related transcription factor 2;

NM_002581 – PAPPA, pregnancy-associated plasma protein A, pappalysin 1;

1. **Genes induced at least 1.5-fold exclusively in differentiated HepaRG cells (84 genes).**

NM_006504 /// NM_130435 – PTPRE, protein tyrosine phosphatase, receptor type, E;

NM_052947 - ALPK2, alpha-kinase 2;

NM_001145160 /// NM_003290 /// XR_016808 /// XR_019021 /// XR_039579 - LOC643634 /// TPM4, similar to tropomyosin 4 /// tropomyosin 4

NM_203394 - E2F7, E2F transcription factor 7;

NM_001159651 /// NM_138369 - BOD1, biorientation of chromosomes in cell division 1;

NM_015205 /// NM_032189 - ATP11A, ATPase, class VI, type 11A

NM_001116 - ADCY9, adenylate cyclase 9;

NM_001145657 /// NM_001145658 /// NM_002885 - RAP1GAP, RAP1 GTPase activating protein;

NM_002133 - HMOX1, heme oxygenase (decycling) 1;

NM_015158 /// NM_153186 - KANK1, KN motif and ankyrin repeat domains 1;

NM_002198 - IRF1, interferon regulatory factor 1;

NM_001145014 - RFPL4A, ret finger protein-like 4A;

NM_152594 - SPRED1, sprouty-related, EVH1 domain containing 1;

NM_205855 - FAM180A, family with sequence similarity 180, member A;

NM_002089 - CXCL2, chemokine (C-X-C motif) ligand 2;

NM_001789 /// NM_201567 - CDC25A, cell division cycle 25 homolog A (S. pombe);

NM_001039667 /// NM_139314 - ANGPTL4, angiopoietin-like 4;

NM_017655 - GIPC2, GIPC PDZ domain containing family, member 2;

NM_001030287 /// NM_001040619 /// NM_001674 /// NM_004024 - ATF3, activating transcription factor 3

NM_145804 - ABTB2, ankyrin repeat and BTB (POZ) domain containing 2;

NM_022486 - SUSD1, sushi domain containing 1;

NM_016095 - GINS2, GINS complex subunit 2 (Psf2 homolog);

NM_001145206 - KIAA1671, KIAA1671;

NM_001024215 /// NM_001024216 /// NM_017556 - FBLIM1, filamin binding LIM protein 1;

NM_001025242 /// NM_001025243 /// NM_001569 - IRAK1, interleukin-1 receptor-associated kinase 1;

NM_004995 - MMP14, matrix metallopeptidase 14 (membrane-inserted);

NM_000124 /// NM_170753 - ERCC6 /// PGBD3, excision repair cross-complementing rodent repair deficiency, complementation group;

NM_001001890 /// NM_001122607 /// NM_001754 - RUNX1, runt-related transcription factor 1;

NM_024094 - DSCC1, defective in sister chromatid cohesion 1 homolog (S. cerevisiae);

NM_001123383 /// NM_001123384 /// NM_001123385 /// NM_017745 – BCOR, BCL6 co-repressor;

NM_001486 – GCKR, glucokinase (hexokinase 4) regulator;

NM_152716 - PATL1, protein associated with topoisomerase II homolog 1 (yeast);

NM_004864 - GDF15, growth differentiation factor 15;

NM_014521 - SH3BP4, SH3-domain binding protein 4;

NM_000480 /// NM_001025389 /// NM_001025390 - AMPD3, adenosine monophosphate deaminase (isoform E);

NM_001164281 /// NM_001164282 /// NM_001164283 /// NM_147200 /// NM_147686 - TRAF3IP2, TRAF3 interacting protein 2;

NM_017858 – TIPIN, TIMELESS interacting protein;

NM_001031804 /// NM_005360 – MAF, v-maf musculoaponeurotic fibrosarcoma oncogene homolog (avian);

NM_001254 - CDC6, cell division cycle 6 homolog (S. cerevisiae);

NM_020182 /// NM_199169 /// NM_199170 /// NM_199171 - PMEPA1, prostate transmembrane protein, androgen induced 1;

NM_001008710 /// NM_001008711 /// NM_001008712 /// NM_006867 – RBPMS, RNA binding protein with multiple splicing;

NM_005239 - ETS2, v-ets erythroblastosis virus E26 oncogene homolog 2 (avian);

NM_001098424 /// NM_004087 - DLG1, discs, large homolog 1 (Drosophila);

NM_005914 /// NM_182746 - MCM4, minichromosome maintenance complex component 4;

NM_001451 - FOXF1, forkhead box F1;

NM_032206 - NLRC5, NLR family, CARD domain containing 5;

NM_000576 - IL1B, interleukin 1, beta;

NM_006442 - DRAP1, DR1-associated protein 1 (negative cofactor 2 alpha);

NM_020133 - AGPAT4, 1-acylglycerol-3-phosphate O-acyltransferase 4 (lysophosphatidic acid acyltransferase;

NM_001020818 /// NM_001020819 /// NM_001020820 /// NM_001020821 /// NM_138373 – MYADM, myeloid-associated differentiation marker;

NM_014325 - CORO1C, coronin, actin binding protein, 1C;

NM_004827 - ABCG2, ATP-binding cassette, sub-family G (WHITE), member 2;

NM_001102654 /// NM_002527 - NTF3, neurotrophin 3;

NM_015482 /// NM_021945 - SLC22A23, solute carrier family 22, member 23;

NM_006290 - TNFAIP3, tumor necrosis factor, alpha-induced protein 3;

NM_003648 /// NM_152879 – DGKD, diacylglycerol kinase, delta 130kDa;

NM_032515 – BOK, BCL2-related ovarian killer;

NM_001037131 /// NM_014914 - AGAP1, ArfGAP with GTPase domain, ankyrin repeat and PH domain 1;

NM_004526 - MCM2, minichromosome maintenance complex component 2;

NM_002577 /// NR_027053 - LOC646214 /// PAK2, p21-activated kinase 2 pseudogene /// p21 protein (Cdc42/Rac)-activated kinase 2;

NM_152718 – VWCE, von Willebrand factor C and EGF domains;

NM_138370 - SGK493, protein kinase-like protein SgK493;

NM_007350 - PHLDA1, pleckstrin homology-like domain, family A, member 1;

NM_018518 /// NM_182751 - MCM10, minichromosome maintenance complex component 10;

NM_005792 - MPHOSPH6, M-phase phosphoprotein 6;

NM_016448 – DTL, denticleless homolog (Drosophila);

NM_000376 /// NM_001017535 – VDR, vitamin D (1,25- dihydroxyvitamin D3) receptor;

NM_006598 - SLC12A7, solute carrier family 12 (potassium/chloride transporters), member 7;

NM_003943 - STBD1, starch binding domain 1;

NM_002916 /// NM_181573 - RFC4, replication factor C (activator 1) 4, 37kDa;

NM_022748 - TNS3, tensin 3;

NM_024310 - PLEKHF1, pleckstrin homology domain containing, family F (with FYVE domain) member 1;

NM_003174 /// NM_021738 – SVIL, supervillin;

NM_005178 - BCL3, B-cell CLL/lymphoma 3;

NM_000584 - IL8, interleukin 8;

NM_001011666 /// NM_004904 /// NM_182898 /// NM_182899 - CREB5, cAMP responsive element binding protein 5;

NM_001114121 /// NM_001114122 /// NM_001274 - CHEK1, CHK1 checkpoint homolog (S. pombe);

NM_025225 - PNPLA3, patatin-like phospholipase domain containing 3;

NM_001139466 /// NM_001139467 /// NM_001139468 /// NM_005647 - TBL1X, transducin (beta)-like 1X-linked;

NM_152308 - C16orf75, chromosome 16 open reading frame 75;

NM_020165 - RAD18, RAD18 homolog (S. cerevisiae);

NM_152906 - C22orf25, chromosome 22 open reading frame 25;

NM_001048201 /// NM_013282 - UHRF1, ubiquitin-like with PHD and ring finger domains 1;

NM_006382 /// NM_031456 - CDRT1 /// FBXW10, CMT1A duplicated region transcript 1 /// F-box and WD repeat domain containing 1;

1. **Genes induced at least 1.5-fold exclusively in undifferentiated HepaRG cells (118 genes).**

NM_013370 /// NM_182980 /// NM_182981 - OSGIN1, oxidative stress induced growth inhibitor 1;

NM_001031628 /// NM_001033873 – SMAGP, small trans-membrane and glycosylated protein;

NM_004235 - KLF4, Kruppel-like factor 4 (gut);

NM_001040610 /// NM_024580 - EFTUD1, elongation factor Tu GTP binding domain containing 1;

NM_001037125 /// NM_023076 – UNKL, unkempt homolog (Drosophila)-like;

NM_018410 – HJURP, Holliday junction recognition protein;

NM_004864 /// XM_002345162 - GDF15 /// LOC100292463, growth differentiation factor 15 /// similar to growth differentiation factor 15;

NM_001432 – EREG, epiregulin;

NM_014747 - RIMS3, regulating synaptic membrane exocytosis 3;

NM_002189 /// NM_172200 - IL15RA, interleukin 15 receptor, alpha;

NM_033058 /// NM_184085 /// NM_184086 /// NM_184087 - TRIM55, tripartite motif-containing 55;

NM_025130 - HKDC1, hexokinase domain containing 1;

NM_001498 – GCLC, glutamate-cysteine ligase, catalytic subunit;

NM_012280 /// NM_177434 /// NM_177439 - FTSJ1, FtsJ homolog 1 (E. coli);

NM_001134851 /// NM_001134852 /// NM_003202 /// NM_201632 /// NM_201633 - TCF7, transcription factor 7 (T-cell specific, HMG-box);

NM_001011649 /// NM_018249 - CDK5RAP2, CDK5 regulatory subunit associated protein 2;

NM_000903 /// NM_001025433 /// NM_001025434 - NQO1, NAD(P)H dehydrogenase, quinone 1;

NM_001037330 /// NM_006470 - TRIM16 /// TRIM16L, tripartite motif-containing 16 /// tripartite motif-containing 16-like;

NM_001017963 /// NM_005348 - HSP90AA1 /// HSP90AA4P, heat shock protein 90kDa alpha (cytosolic), class A member 1;

NM_002526 - NT5E, 5'-nucleotidase, ecto (CD73);

NM_001161452 /// NM_001161454 /// NM_153611 - CYBASC3, cytochrome b, ascorbate dependent 3;

NM_002053 - GBP1, guanylate binding protein 1, interferon-inducible, 67kDa;

NM_024420 - PLA2G4A, phospholipase A2, group IVA (cytosolic, calcium-dependent);

NM_002203 - ITGA2, integrin, alpha 2 (CD49B, alpha 2 subunit of VLA-2 receptor);

NM_016084 - RASD1, RAS, dexamethasone-induced 1;

NM_022825 /// NM_203473 /// NM_203474 /// NM_203475 /// NM_203476 – PORCN, porcupine homolog (Drosophila);

NM_004973 - JARID2, jumonji, AT rich interactive domain 2;

NM_006142 – SFN, stratifin;

NM_017669 - ERCC6L, excision repair cross-complementing rodent repair deficiency, complementation group;

NM_001008707 /// NM_004434 - EML1, echinoderm microtubule associated protein like 1;

NM_003607 /// NM_014826 - CDC42BPA, CDC42 binding protein kinase alpha (DMPK-like);

NM_002748 - MAPK6, mitogen-activated protein kinase 6;

NM_014568 - GALNT5, UDP-N-acetyl-alpha-D-galactosamine:polypeptide N-acetylgalactosaminyltransferase;

NM_000745 - CHRNA5, cholinergic receptor, nicotinic, alpha 5;

NM_001146319 /// NM_001146320 /// NM_001146321 /// NM_001146322 /// NM_023927 - GRAMD3, GRAM domain containing 3;

NM_001144955 /// NM_020234 - DTWD1, DTW domain containing 1;

NM_001007538 - SHISA2, shisa homolog 2 (Xenopus laevis);

NM_007107 - SSR3, signal sequence receptor, gamma (translocon-associated protein gamma);

NM_014689 - DOCK10, dedicator of cytokinesis 10;

NM_006855 /// NM_016657 - KDELR3, KDEL (Lys-Asp-Glu-Leu) endoplasmic reticulum protein retention receptor 3;

NM_001018054 /// NM_004631 /// NM_017522 /// NM_033300 - LRP8, low density lipoprotein receptor-related protein 8, apolipoprotein e receptor;

NM_173529 - C18orf54, chromosome 18 open reading frame 54;

NM_000103 /// NM_031226 - CYP19A1, cytochrome P450, family 19, subfamily A, polypeptide 1;

NM_014226 – RAGE, renal tumor antigen;

NM_013451 /// NM_133337 – MYOF, myoferlin;

NM_022779 /// NM_138620 - DDX31, DEAD (Asp-Glu-Ala-Asp) box polypeptide 31;

NM_022361 /// NR_024539 - POPDC3, popeye domain containing 3;

NM_001143995 /// NM_004811 – LPXN, leupaxin;

NM_021101 - CLDN1, claudin 1;

NM_032146 /// NM_177976 - ARL6, ADP-ribosylation factor-like 6;

NM_003483 /// NM_003484 - HMGA2, high mobility group AT-hook 2;

NM_080725 - SRXN1;

NM_017998 - C9orf40, chromosome 9 open reading frame 40;

NM_005438 - FOSL1, FOS-like antigen 1;

NM_001145160 /// NM_003290 - TPM4, tropomyosin 4;

NM_001032283 /// NM_001032284 /// NM_003276 – TMPO, thymopoietin;

NM_000602 - SERPINE1, serpin peptidase inhibitor, clade E (nexin, plasminogen activator inhibitor type 1)

NM_000433 /// NM_001127651 - NCF2, neutrophil cytosolic factor 2;

NM_001881 /// NM_181571 /// NM_182717 /// NM_182718 /// NM_182719 /// NM_182720 – CREM, cAMP responsive element modulator;

NM_001134405 /// NM_001134406 /// NM_138290 - RUNDC3B, RUN domain containing 3B;

NM_001102559 /// NM_001102560 /// NM_032483 - PPAPDC1B, phosphatidic acid phosphatase type 2 domain containing 1B;

NM_005242 - F2RL1, coagulation factor II (thrombin) receptor-like 1;

NM_014547 - TMOD3, tropomodulin 3 (ubiquitous);

NM_001969 /// NM_183004 - EIF5, eukaryotic translation initiation factor 5;

NM_001018115 /// NM_033084 - FANCD2, Fanconi anemia, complementation group D2;

NM_014783 /// NM_199357 - ARHGAP11A, Rho GTPase activating protein 11A;

NM_004237 - TRIP13, thyroid hormone receptor interactor 13;

NM_000574 /// NM_001114752 - CD55, CD55 molecule, decay accelerating factor for complement (Cromer blood group);

NM_001005376 /// NM_001005377 /// NM_002659 – PLAUR, plasminogen activator, urokinase receptor;

NM_001017424 /// NM_001017425 /// NM_014217 - KCNK2, potassium channel, subfamily K, member 2;

NM_001024666 /// NM_031892 - SH3KBP1, SH3-domain kinase binding protein 1;

NM_000691 /// NM_001135167 /// NM_001135168 - ALDH3A1, aldehyde dehydrogenase 3 family, memberA1;

NM_003507 - FZD7, frizzled homolog 7 (Drosophila);

NM_145018 - C11orf82, chromosome 11 open reading frame 82;

NM_001236 - CBR3, carbonyl reductase 3;

NM_001134367 /// NM_001134368 /// NM_003043 - SLC6A6, solute carrier family 6 (neurotransmitter transporter, taurine), member 6;

NM_002890 /// NM_022650 - RASA1, RAS p21 protein activator (GTPase activating protein) 1;

NM_015566 - FAM169A, family with sequence similarity 169, member A;

NM_003359 – UGDH, UDP-glucose dehydrogenase;

NM_001039535 /// NM_145060 - SKA1, spindle and kinetochore associated complex subunit 1;

NM_173582 - PGM2L1, phosphoglucomutase 2-like 1;

NM_001145319 /// NM_002670 - PLS1, plastin 1 (I isoform);

NM_001099691 /// NM_003236 – TGFA, transforming growth factor, alpha;

NM_020166 - MCCC1, methylcrotonoyl-Coenzyme A carboxylase 1 (alpha);

NM_018124 - RFWD3, ring finger and WD repeat domain 3;

NM_006154 /// NM_198400 - NEDD4, neural precursor cell expressed, developmentally down-regulated 4;

NM_000463 /// NM_001072 /// NM_007120 /// NM_019075 /// NM_019076 /// NM_019077 - UGT1A1 /// UGT1A10 /// UGT1A3 /// UGT1A4 /// UGT1A5 /// UGT1A6 /// UGT1A7 /// UGT1A8 /// UGT1A9, UDP glucuronosyltransferase 1 family, polypeptide A1-A10;

NM_014822 - SEC24D, SEC24 family, member D (S. cerevisiae);

NM_001146276 /// NM_001146277 /// NM_001146278 /// NM_020792 - NCEH1, neutral cholesterol ester hydrolase 1;

NM_000685 /// NM_004835 /// NM_009585 /// NM_031850 /// NM_032049 - AGTR1, angiotensin II receptor, type 1;

NM_001004298 - C10orf90, chromosome 10 open reading frame 90;

NM_020918 – GPAM, glycerol-3-phosphate acyltransferase, mitochondrial;

NM_025217 - ULBP2, UL16 binding protein 2;

NM_001031848 /// NM_002640 /// NM_198833 - SERPINB8, serpin peptidase inhibitor, clade B (ovalbumin), member 8;

NM_014059 - C13orf15, chromosome 13 open reading frame 15;

NM_000544 /// NM_018833 - TAP2, transporter 2, ATP-binding cassette, sub-family B (MDR/TAP);

NM_000169 – GLA, galactosidase, alpha;

NM_005813 - PRKD3, protein kinase D3;

NM_017709 - FAM46C, family with sequence similarity 46, member C;

NM_001657 – AREG, amphiregulin;

NM_015440 - MTHFD1L, methylenetetrahydrofolate dehydrogenase (NADP+ dependent) 1-like;

NM_018284 - GBP3, guanylate binding protein 3;

NM_007193 - ANXA10, annexin A10;

NM_001099652 - GPR137C, G protein-coupled receptor 137C;

NM_000596 - IGFBP1, insulin-like growth factor binding protein 1;

NM_013247 /// NM_145074 - HTRA2, HtrA serine peptidase 2;

NM_015986 - CRLF3, cytokine receptor-like factor 3;

NM_138969 - SDR16C5, SDR16C5;

NM_016464 - TMEM138, transmembrane protein 138;

NM_020947 - KIAA1609, KIAA1609;

NM_002061 – GCLM, glutamate-cysteine ligase, modifier subunit;

NM_006547 - IGF2BP3, insulin-like growth factor 2 mRNA binding protein 3;

NM_005112 /// NM_017491 - WDR1, WD repeat domain 1;

NM_014363 – SACS, spastic ataxia of Charlevoix-Saguenay (sacsin);

NM_001079516 /// NM_001143974 /// NM_019893 /// XM_927086 - ASAH2 /// ASAH2B /// ASAH2C, N-acylsphingosine amidohydrolase (non-lysosomal ceramidase) 2;

NM_005842 - SPRY2, sprouty homolog 2 (Drosophila);

NM_002820 /// NM_198964 /// NM_198965 /// NM_198966 – PTHLH, parathyroid hormone-like hormone;

NM_017975 /// NR_003105 – ZWILCH, Zwilch, kinetochore associated, homolog (Drosophila);
