# Supplementary material for: Induction of expression of aryl hydrocarbon receptor-dependent genes in human HepaRG cell line modified by shRNA and treated with β-naphthoflavone
Source: Mol Cell Biochem. 2016 Oct 28;425(1):59–75. doi: 10.1007/s11010-016-2862-3 (PMC5225230; doi:10.1007/s11010-016-2862-3)
Supplement: Supplementary file 3 — Supplementary material 3 (DOCX 22 kb) [file 11010_2016_2862_MOESM3_ESM.docx]

List of genes inhibited in AhR dependent manner at least 1.5-fold (P≤0.05) by BNF treatment of undifferentiated and differentiated HepaRG cells. Data presented in Fig.2.

1. **Genes inhibited in both, undifferentiated as well as differentiated HepaRG cells (94 genes).**

NM_001134670 /// NM_138413 – DHDPSL, dihydrodipicolinate synthase-like, mitochondrial

NM_001142861 /// NM_005585 /// NR_027654 - SMAD6, SMAD family member 6

NM_001001852 - PIM3, pim-3 oncogene

NM_001442 - FABP4, fatty acid binding protein 4, adipocyte

NM_001142883 /// NM_054111 - IP6K3, inositol hexakisphosphate kinase 3

NM_016205 – PDGFC, platelet derived growth factor C

NM_002705 – PPL, periplakin

NM_001013642 - TRNP1, TMF1-regulated nuclear protein 1

NM_001114661 /// NM_006226 - PLCL1, phospholipase C-like 1

NM_001085486 /// NM_001093726 /// NM_005410 - SEPP1, selenoprotein P, plasma, 1;

NM_138569 - C6orf142, chromosome 6 open reading frame 142;

NM_001024074 /// NM_001024075 /// NM_006895 – HNMT, histamine N-methyltransferase;

NM_000240 – MAOA, monoamine oxidase A;

NM_021111 – RECK, reversion-inducing-cysteine-rich protein with kazal motifs;

NM_005764 - PDZK1IP1, PDZK1 interacting protein 1;

NM_014899 - RHOBTB3, Rho-related BTB domain containing 3;

NM_019035 - PCDH18, protocadherin 18;

NM_001134848 - CCDC152, coiled-coil domain containing 152;

NM_000689 - ALDH1A1, aldehyde dehydrogenase 1 family, member A1;

NM_005164 - ABCD2, ATP-binding cassette, sub-family D (ALD), member 2;

NM_019050 - USP53, ubiquitin specific peptidase 53;

NM_014079 - KLF15, Kruppel-like factor 15;

NM_152328 /// NM_199165 - ADSSL1, adenylosuccinate synthase like 1;

NM_001850 /// NM_020351 - COL8A1, collagen, type VIII, alpha 1;

NM_001393 - ECM2, extracellular matrix protein 2, female organ and adipocyte specific;

NM_207312 - TUBA3E, tubulin, alpha 3e;

NM_000165 - GJA1, gap junction protein, alpha 1, 43kDa;

NM_005141 – FGB, fibrinogen beta chain;

NM_001005242 /// NM_004572 - PKP2, plakophilin 2;

NM_014585 - SLC40A1, solute carrier family 40 (iron-regulated transporter), member 1;

NM_021205 – RHOU, ras homolog gene family, member U;

NM_182493 - MYLK3, myosin light chain kinase 3;

NM_001145453 /// NM_005264 /// NM_145793 - GFRA1, GDNF family receptor alpha 1;

NM_000248 /// NM_006722 /// NM_198158 /// NM_198159 /// NM_198177 /// NM_198178 – MITF, microphthalmia-associated transcription factor;

NM_000055 – BCHE, butyrylcholinesterase;

NM_003155 - STC1, stanniocalcin 1;

NM_001161661 /// NM_001161662 /// NM_015238 - WWC1, WW and C2 domain containing 1;

NM_004975 - KCNB1, potassium voltage-gated channel, Shab-related subfamily, member 1;

NM_001105250 /// NM_004796 /// NM_138970 - NRXN3, neurexin 3;

NM_006166 – NFYB, nuclear transcription factor Y, beta;

NM_000204 – CFI, complement factor I;

NM_001162893 /// NM_001162894 /// NM_001162895 /// NM_014656 /// NR_015350 - KIAA0040, KIAA0040;

NM_183240 - TMEM37, transmembrane protein 37;

NM_020459 - PAIP2B, poly(A) binding protein interacting protein 2B;

NM_002905 - RDH5, retinol dehydrogenase 5 (11-cis/9-cis);

NM_001080433 - CCDC85A, coiled-coil domain containing 85A;

NM_000777 - CYP3A5, cytochrome P450, family 3, subfamily A, polypeptide 5;

NM_054110 - GALNTL2, UDP-N-acetyl-alpha-D-galactosamine:polypeptide N-acetylgalactosaminyltransferase;

NM_015621 - CCDC69, coiled-coil domain containing 69;

NM_003881 - WISP2, WNT1 inducible signaling pathway protein 2;

NM_001034850 /// NM_019000 - FAM134B, family with sequence similarity 134, member B;

NM_006080 - SEMA3A, sema domain, immunoglobulin domain (Ig), short basic domain, secreted;

NM_002276 - KRT19, keratin 19;

NM_173075 - APBB2, amyloid beta (A4) precursor protein-binding, family B, member 2;

NM_014722 /// NM_015864 - FAM65B, family with sequence similarity 65, member B;

NM_001034954 /// NM_001034955 /// NM_001034956 /// NM_001034957 /// NM_006434 - SORBS1, sorbin and SH3 domain containing 1;

NM_004432 - ELAVL2, ELAV (embryonic lethal, abnormal vision, Drosophila)-like 2 (Hu antigen B);

NM_181504 /// NM_181523 /// NM_181524 - PIK3R1, phosphoinositide-3-kinase, regulatory subunit 1 (alpha);

NM_001885 – CRYAB, crystallin, alpha B;

NM_000900 – MGP, matrix Gla protein;

NM_152395 - NUDT16, nudix (nucleoside diphosphate linked moiety X)-type motif 16;

NM_012190 - ALDH1L1, aldehyde dehydrogenase 1 family, member L1;

NM_001979 - EPHX2, epoxide hydrolase 2, cytoplasmic;

NM_001099855 /// NM_005369 - MCF2, MCF.2 cell line derived transforming sequence;

NM_001172 - ARG2, arginase, type II;

NM_001099677 /// NM_020844 - C8orf79, chromosome 8 open reading frame 79;

NM_080386 - TUBA3D, tubulin, alpha 3d;

NM_001742 – CALCR, calcitonin receptor;

NM_013281 /// NM_198391 - FLRT3, fibronectin leucine rich transmembrane protein 3;

NM_001025604 /// NM_015683 - ARRDC2, arrestin domain containing 2;

NM_153345 - TMEM139, transmembrane protein 139;

NM_152423 - MUM1L1, melanoma associated antigen (mutated) 1-like 1;

NM_006877 – GMPR, guanosine monophosphate reductase;

NM_006100 - ST3GAL6, ST3 beta-galactoside alpha-2,3-sialyltransferase 6;

NM_001105533 /// NM_024913 - C7orf58, chromosome 7 open reading frame 58;

NM_012242 - DKK1, dickkopf homolog 1 (Xenopus laevis);

NM_001003683 /// NM_005019 - PDE1A, phosphodiesterase 1A, calmodulin-dependent;

NM_000779 /// NM_001099772 - CYP4B1, cytochrome P450, family 4, subfamily B, polypeptide 1;

NM_004205 /// NM_171997 - USP2, ubiquitin specific peptidase 2;

NM_012301 - MAGI2, membrane associated guanylate kinase, WW and PDZ domain containing 2;

NM_022094 – CIDEC, cell death-inducing DFFA-like effector c;

NM_001076680 - LOC201229, hypothetical protein LOC201229;

NM_003392 - WNT5A, wingless-type MMTV integration site family, member 5A;

NM_001901 – CTGF, connective tissue growth factor;

NM_001005473 - PLCXD3, phosphatidylinositol-specific phospholipase C, X domain containing 3,

NM_017762 - MTMR10, myotubularin related protein 10;

NM_000724 /// NM_201570 /// NM_201571 /// NM_201572 /// NM_201590 /// NM_201593 - CACNB2, calcium channel, voltage-dependent, beta 2 subunit;

NM_032902 - PPP1R16A, protein phosphatase 1, regulatory (inhibitor) subunit 16A;

NM_001002294 /// NM_006894 - FMO3, Flavin containing monooxygenase 3;

NM_002253 – KDR, kinase insert domain receptor (a type III receptor tyrosine kinase);

NM_014840 - NUAK1, NUAK family, SNF1-like kinase, 1;

NM_001031716 /// NR_024415 - OBFC2A, oligonucleotide/oligosaccharide-binding fold containing 2A;

NM_016201 - AMOTL2, angiomotin like 2

NM_002614 /// NR_003377 - PDZK1 /// PDZK1P1, similar to PDZ domain containing 1 /// PDZ domain containing 1

1. **Genes inhibited at least 1.5-fold exclusively in differentiated HepaRG cells (104 genes).**

NM_006887

NM_001206

NM_145740;

NM_014606;

NM_000846;

NM_001080538;

NM_000477;

NM_024621;

NM_003273;

NM_058229 /// NM_148177;

NM_198993;

NM_022449;

NM_018342;

NM_001001787 /// NM_001677;

NM_001753;

NM_000667 /// NM_000668 /// NM_000669;

NM_018710;

NM_020152;

NM_152618;

NM_001145311 /// NM_002666;

NM_001039538 /// NM_002374 /// NM_031845 /// NM_031847;

NM_138420;

NM_000668;

NM_203463;

NM_025194;

NM_014510 /// NM_033026;

NM_001127671 /// NM_002310;

NM_004285;

NM_138288;

NM_001002258 /// NM_001689;

NM_006343;

NM_001105529 /// NM_006095;

NM_152542;

NM_001098540 /// NM_006665;

NM_001608

NM_001047160 /// NM_005863

NM_001005914 /// NM_004636

NM_001627

NM_005763

NM_020299

NM_002026 /// NM_054034 /// NM_212474 /// NM_212475 /// NM_212476 /// NM_212478

NM_004768

NM_001080538 /// NM_020299 /// XR_016984 /// XR_018726 /// XR_038524

NM_001024844 /// NM_002231

NM_152637

NM_001122606 /// NM_002294 /// NM_013995

NM_000950 /// NM_001142395

NM_006419

NM_001134285 /// NM_001438 /// NM_206594 /// NM_206595 /// NR_024099

NM_017460

NM_001015045 /// NM_014883

NM_007312 /// NM_033159 /// NM_153281 /// NM_153282 /// NM_153283 /// NM_153285

NM_003944

NM_000582 /// NM_001040058 /// NM_001040060

NM_024900 /// NM_199320

NM_001098512 /// NM_006258

NM_001128304 /// NM_001128305 /// NM_001128306 /// NM_020353

NM_001002810 /// NM_001002811 /// NM_001002812 /// NM_014644 /// NM_022359

NM_014141

NM_000899 /// NM_003994

NM_000680 /// NM_033302 /// NM_033303 /// NM_033304

NM_012158

NM_001127174 /// NM_001127175 /// NM_001127176 /// NM_031939

NM_021038 /// NM_207292 /// NM_207293 /// NM_207294 /// NM_207295 /// NM_207296

NM_019117 /// NM_057162

NM_004318 /// NM_020164 /// NM_032466 /// NM_032467 /// NM_032468

NM_001872 /// NM_016413

NM_001562

NM_021153

NM_001002233 /// NM_001002814 /// NM_025151

NM_001001994 /// NM_001001995 /// NM_001001996 /// NM_005278

NM_000346

NM_001130688 /// NM_001130689 /// NM_002129

NM_001146

NM_207336

NM_001130715 /// NM_001130716 /// NM_016619

NM_144650

NM_005589

NM_030821

NM_002214

NM_031372 /// NR_003249

NM_016567 /// NM_078468 /// NM_078469

NM_001012339 /// NM_194283

NM_018344

NM_001040152 /// NM_015068

NM_153486 /// NM_194436

NM_001143824 /// NM_018018

NM_000896

NM_004815

NM_001159

NM_015319 /// NM_170754 /// NM_198316

NM_015267

NM_018208

NM_005398

NM_001424

NM_002803

NM_004368 /// NM_201277

NM_002273

NM_001145775 /// NM_001145776 /// NM_001145777 /// NM_004117

NM_014900

NM_001954 /// NM_013993 /// NM_013994

NM_006379

NM_001233 /// NM_198212

NM_015199

1. **Genes inhibited at least 1.5-fold exclusively in undifferentiated HepaRG cells (161 genes)**.

NM_001935

NM_003621

NM_139283

NM_016206

NM_003107

NM_000694 /// NM_001030010 /// NM_001161473

NM_001143996 /// NM_001143997 /// NM_018245

NM_001135820 /// NM_013390

NM_006308

NM_001134437 /// NM_001134438 /// NM_001134439 /// NM_145753

NM_001039348 /// NM_001039349 /// NM_004105

NM_001130083 /// NM_001130084 /// NM_001130085 /// NM_001130086 /// NM_001130087

NM_004938

NM_014505

NM_001134673 /// NM_001145511 /// NM_001145512 /// NM_005595

NM_001033049 /// NM_001112 /// NM_001145407 /// NM_001160230 /// NM_015833

NM_014631

NM_000062 /// NM_001032295

NM_014646

NM_001076552 /// NM_018677 /// NR_028046

NM_002959

NM_001142397 /// NM_014772

NM_173462

NM_001145398 /// NM_003216

NM_002272

NM_002841

NM_018030 /// NM_080597

NM_001126105 /// NM_001126106 /// NM_003982

NM_025179

NM_006294

NM_000155

NM_001145026

NM_002185

NM_000063 /// NM_001145903 /// NM_001710

NM_002704

NM_001142270 /// NM_001142271 /// NM_005771 /// NM_199204

NM_021785

NM_017823

NM_000216

NM_017644

NM_001031700 /// NM_001128424 /// NM_016613

NM_000508 /// NM_021871

NM_025149

NM_005554 /// NM_005555 /// NM_173086

NM_018988

NM_001124

NM_000163

NM_003278

NM_003562

NM_006076

NM_001030060

NM_012431

NM_000901

NM_018421

NM_005234

NM_003243

NM_002546

NM_001130016 /// NM_001130017 /// NM_001179

NM_002012

NM_052972

NM_001126337 /// NM_020127

NM_001099640 /// NM_018286

NM_005560

NM_015150

NM_014452

NM_016335

NM_031430

NM_198503

NM_014315

NM_001632

NM_007207 /// NM_144728 /// NM_144729

NM_000041

NM_032023

NM_001129981 /// NM_020349

NM_002015

NM_025074

NM_005276

NM_001102366 /// NM_001102367 /// NM_001102368 /// NM_001102369 /// NM_024952

NM_024560

NM_000591 /// NM_001040021

NM_001017523 /// NM_001018072

NM_001115

NM_001136007 /// NM_001136008 /// NM_001136009 /// NM_001136010 /// NM_001136011

NM_004655

NM_145693

NM_001080400

NM_001042625 /// NM_144647

NM_000264 /// NM_001083602 /// NM_001083603 /// NM_001083604 /// NM_001083605

NM_014974

M_000331 /// NM_001127380 /// NM_030754 /// NM_199161

NM_003743 /// NM_147223 /// NM_147233

NM_001482

NM_013324 /// NM_145071

NM_001145524 /// NM_031477

XR_042106 /// XR_042107

NM_001136494 /// NM_001136495 /// NM_032800

NM_000638 /// NM_001080837 /// NM_001083896

NM_001003818 /// NM_058166

NM_032812

NM_001548

NM_000509 /// NM_021870

NM_022165

NM_015345

NM_006079

NM_005277 /// NM_201591 /// NM_201592

NM_032693

NM_001099781 /// NM_001099782 /// NM_004121

NM_001003792 /// NM_001003793 /// NM_014483

NM_024007

NM_006646

NM_003204

NM_001343

NM_006499 /// NM_201543 /// NM_201544 /// NM_201545

NM_000141 /// NM_001144913 /// NM_001144914 /// NM_001144915 /// NM_001144916

NM_013261

NM_000908

NM_001032278 /// NM_024302

NM_001143805 /// NM_001143806 /// NM_001143807 /// NM_001143808 /// NM_001143809

NM_001144881 /// NM_001144882 /// NM_001144883 /// NM_153026

NM_001098518 /// NM_015234

NM_199051

NM_001455 /// NM_201559

NM_007085

NM_000902 /// NM_007287 /// NM_007288 /// NM_007289

NM_001831 /// NM_203339

NM_024607

NM_021977

NM_004560

NM_013238

NM_001015880 /// NM_004670

NM_032564

NM_001083 /// NM_033430 /// NM_033437

NM_003247

NM_014859

NM_178565

NM_006515 /// NR_024022

NM_000015

NM_004428 /// NM_182685

NM_001005404

NM_018214

NM_001127380 /// NM_030754

NM_005165

NM_015429

NM_001029851 /// NM_001029852 /// NM_001029853 /// NM_001029854 /// NM_003719

NM_001009571 /// NM_017954

NM_015177

NM_006512

NM_198353

NM_004451

NM_001099625 /// NM_001099626 /// NM_001099627 /// NM_019557

NM_022153

NM_017734

NM_024563

NM_006775 /// NM_206853 /// NM_206854 /// NM_206855

NM_002961 /// NM_019554

NM_001079855 /// NM_003918

NM_001128615 /// NM_001128616 /// NM_019555

NM_001455 /// NM_201559 /// NR_026718

NM_020312

NM_020859

NM_001143676 /// NM_001143677 /// NM_001143678 /// NM_005627
